# Histone-Binding Protein RBBP4 Is Necessary to Promote Neurogenesis in the Developing Mouse Neocortical Progenitors

**DOI:** 10.1523/ENEURO.0391-23.2024

**Published:** 2024-11-26

**Authors:** Sreeja Kumari Dhanya, Kishan Kalia, Sattwik Mohanty, Tulaib Azam, Asha S. Channakkar, Leora D’Souza, K.S. Swathi, Puli Chandramouli Reddy, Bhavana Muralidharan

**Affiliations:** 1 Institute for Stem Cell Science and Regenerative Medicine (inStem), Bangalore 560065, India; 2 Regional Centre for Biotechnology, Faridabad 121001, India; 3 CoEE, Department of Life Sciences, School of Natural Sciences, Shiv Nadar Institution of Eminence, Delhi 201314, India

**Keywords:** Cdon, histone-binding, mouse neocortical development, neurogenesis, nucleosome remodeling complex, Polycomb repressive complex, Rbbp4

## Abstract

Chromatin regulation plays a crucial role in neocortical neurogenesis, and mutations in chromatin modifiers are linked to neurodevelopmental disorders. RBBP4 is a core subunit of several chromatin-modifying complexes; however, its functional role and genome-wide occupancy profile in the neocortical primordium are unknown. To address this, we performed RBBP4 knockdown using CRISPR/Cas9 on neocortical progenitors derived from mice of both sexes at embryonic age 12.5 during deep layer neurogenesis. Our study demonstrates that downregulation of RBBP4 in the E12.5 neocortical progenitors reduced neuronal output, specifically affecting CTIP2-expressing neurons. We demonstrate that RBBP4 plays an essential role in regulating neocortical progenitor proliferation. However, overexpression of RBBP4 alone was not sufficient to regulate neuronal fate. Genome-wide occupancy analysis revealed that RBBP4 primarily binds to distal regulatory elements, and neuron differentiation is a significant GO biological pathway of RBBP4-bound genes. Interestingly, we found that RBBP4 binds to *Cdon*, a receptor protein in the Shh signaling pathway, and knockdown of *Cdon* phenocopies RBBP4 knockdown resulting in a significant reduction in neurogenesis, particularly CTIP2-expressing neurons. CDON overexpression could rescue the phenotype caused upon loss of RBBP4 in the neocortex, thereby suggesting the functional link between RBBP4 and its target gene CDON. Our results shed light on the cellular role of RBBP4 and identify CDON as a novel regulator of deep layer neurogenesis in the neocortical progenitors. Our findings are significant in the context of understanding how dysregulated chromatin regulation impacts cellular mechanisms in neurodevelopmental disorders.

## Introduction

The mammalian neocortex is a complex laminar structure consisting of six layers, each comprising specific neuronal subtypes that enable higher-order functions by forming unique functional connections across different regions of the brain. The glutamatergic neurons and glia of the neocortical primordium arise from the ventricular zone (VZ), which comprises the apical radial glia (aRG), also known as the neocortical progenitors (Malatesta et al., 2000; Miyata et al., 2001; Noctor et al., 2002; Kriegstein and Noctor, 2004). Neurogenesis in the cerebral cortex occurs between embryonic day (E) 10.5 to 17.5 and is timed to generate adequate numbers of each neuronal subtype (Rakic, 1988; Caviness et al., 1995; McConnell, 1995; Zhong et al., 2000; Fishell and Kriegstein, 2003). The different layers are generated in an inside-out manner, with the deep layer neurons, namely, corticothalamic projection neurons (CTPNs) and subcerebral projection neurons (SCPNs), generated first, followed by the upper layer neurons (McConnell, 1988; Hartfuss et al., 2001; Malatesta et al., 2003). Chromatin remodelers play a crucial and deterministic role in the generation and differentiation of glutamatergic neurons (Greig et al., 2013; Tuoc et al., 2013a, b; Sokpor et al., 2017, 2018). Single-cell RNA sequencing analysis of the developing mouse neocortical primordium in early-stage apical progenitors E12–13 shows cell-intrinsic programs such as genes involved in gene regulation and chromatin structure are more prominent (Telley et al., 2019). Indeed, several key chromatin remodeling complex subunits are expressed in the developing neocortical primordium and play critical roles in several steps of neural network formation namely progenitor proliferation, neuronal and glial differentiation, axon path finding, and synapse formation and maturation (Greig et al., 2013; Tuoc et al., 2013a, b; Sokpor et al., 2017, 2018).

Two key chromatin remodeling complexes which are expressed and play a critical role during the neocortical primordium development are the nucleosome remodeling and deacetylase (NuRD) complex (Potts et al., 2011; Florio and Huttner, 2014; Knock et al., 2015; Moon et al., 2017; Hoffmann and Spengler, 2019; D’Souza et al., 2021) and the Polycomb repressive complex (PRC; Bracken et al., 2006; Mohn et al., 2008; Hirabayashi et al., 2009; Petracovici and Bonasio, 2021; Bölicke and Albert, 2022). Retinoblastoma binding protein (RBBP4) is a histone chaperone that is part of both the NuRD (Feng and Zhang, 2003) and PRC2 complexes (Kuzmichev et al., 2002; Conway et al., 2018). Its roles include histone binding (Murzina et al., 2008), complex recruitment, and enzyme enhancement (Millard et al., 2016; Chen et al., 2018). RBBP4 is a ubiquitous nuclear protein with significant developmental and disease-related functions. It is upregulated in several human embryonal brain tumors and glial cancers, indicating its role as a repressor (Forbes et al., 2017). Standard RBBP4 knock-out embryos do not survive, while knockdown of RBBP4 in embryonic stem cells leads to the loss of pluripotency and differentiation into mesoendo-dermal lineages (Miao et al., 2020; Huang et al., 2021). In particular, loss of RBBP4 in the zebrafish brain results in neural progenitor apoptosis (Schultz et al., 2018; Schultz-Rogers et al., 2022). Putative risk variants in RBBP4 have been associated with deficits in neuro-cognitive capacities in autism patients (Firth et al., 2009).

Recently, the role of RBBP4/7 in generating GABAergic neurons has been studied. Reduction in RBBP4/7 results in decreased proliferation of progenitors in the VZ, leading to a reduced size of the medial ganglionic eminence and fewer cortical interneurons (Price et al., 2022). RBBP4 was also found to be co-occupied with LSD1 and HDAC2, members of the NuRD complex, and transcription factor LHX2 in the neocortical primordium. This co-occupation occurred on the promoter and distal enhancer element of Sox11, a key determinant of SCPNs fate in the neocortex (Muralidharan et al., 2017a).

However, the functional and molecular role of RBBP4 in the developing neocortical primordium remains unclear. In this study, we elucidate its novel role in neocortical progenitors at E12.5 by employing a CRISPR/Cas9 approach to knock down Rbbp4. Loss of RBBP4 in the neocortical progenitors at E12.5 results in reduced neurogenesis, specifically affecting the generation of Ctip2-expressing neurons. Notably, overexpression of RBBP4 does not impact neurogenesis. Furthermore, we conducted a genomewide occupancy analysis of RBBP4, identifying Cdon as one of its binding targets. Knockdown of *Cdon* also leads to a reduction in the generation of CTIP2-positive neurons, suggesting that CDON functions similarly to RBBP4 in regulating neocortical neurogenesis. CDON overexpression rescued the reduced neurogenesis and altered neuronal subtype specification seen in the RBBP4-deficient neocortical progenitor cultures, thereby suggesting the functional link between RBBP4 and its target gene CDON. Both RBBP4 and CDON function by regulating neocortical progenitor proliferation. Overall, our study sheds light on RBBP4’s novel role in neocortical development and identifies CDON, one of its binding targets, as a novel cortical gene that regulates deep layer neurogenesis.

## Materials and Methods

### Mice

All experimental procedures followed the guidelines prescribed by the Institutional Animal Ethics Committee approved by the Control and Supervision of Experiments on Animals (*CPCSEA*), New Delhi, India. Experiments were done using CDI mice (Charles River Laboratories). CD-2019 timed pregnant female mice were bred and maintained in the NCBS/inStem Animal Care and Resource Centre, Bangalore, India. Embryonic day 0.5 (E0.5) was defined as the mid-day when a vaginal plug was observed. Early-age embryos were staged by somite number and both male and female embryos were used for the experiment.

### RNA-in situ hybridizations

In situ hybridization (ISH) was performed using digoxigenin-labeled RNA probes. Riboprobes were made using digoxigenin-labeled NTPs (Roche, catalog #11277073910). Antisense and sense probes were amplified using T7 Promoter (ATGCTAATACGACTCACTATAGGG). Cryo glue-embedded E12.5 brain sections (20 μm thickness) were collected on precharged slides (Matsunami Platinum Pro Adhesive Glass Slide) using Slee Mev+ cryostat. The sections were dried at 37°C for 2 h and then fixed in 4% PFA for 15 min as previously described (Muralidharan et al., 2017a). The following washing steps were followed sequentially: three washes with 1× PBS for 5 min each, proteinase K (1 μg/ml) in TE buffer treatment at 37°C for 10 min, and postfixation in 4% PFA for 15 min. The sections were hybridized overnight at 70°C with respective probe mailers (in 50% formamide, 5× SSC, 1× SDS). Post hybridization, stringent washes were performed using preheated solution X containing 50% (v/v) formamide, 2× SSC, and 1% (w/v) SDS. Consecutive washes were carried out using 2× SSC and 0.2× SSC for 25 min each. The sections were washed thrice with filtered fresh TBST (Tris-buffered saline with 0.1% Tween 20) for 15 min each and then incubated overnight with anti-digoxigenin antibody tagged with alkaline phosphatase (1:5,000 in TBST and 1% horse serum, Roche, catalog #12486523) at 4°C. Following TBST (four times for 15 min each) and NTMT [NaCl (0.1 M), Tris-Cl (0.1 M), MgCl2 (0.05 M), Tween 20 (0.1%) washes (10 min)], the antibody was detected using the substrate NBT/BCIP (Roche, 4-nitroblue tetrazolium chloride, catalog #70210625; 5-bromo-4-chloro-3-idolyl phosphate, catalog #70251721). Slides were counterstained with FAST RED (Sigma) coverslipped using DPX mountant and imaged. Probes for *Rbbp4* and *Cdon* were generated using the PCR primers as shown in Table 1 (5′ to 3′). At least three biological replicates were performed for each marker.

### In utero electroporation

Timed pregnant CD1 mice were used for in utero electroporation at E13. Animals were anaesthetized with isoflurane (2% induction, 1.5% during the surgery) and the uterine horns were exposed. Plasmids were used at the total concentration of 2 μg/μl and mixed with Fast Green dye (Sigma, catalog #F7252-5G). For controls, the pX458-Dual-Guide-Donor-Cas9-H2B-mCherry vector was used and for RBBP4 knock down, guide RNAs (gRNA1 and gRNA2) against RBBP4 cloned into pX458-Dual-Guide-Donor-Cas9-H2B-mCherry was used. Approximately 1–3 μl of plasmid was injected into the lateral ventricle of embryos using a pulled glass capillary. Embryos were then electroporated by placing the head of each embryo between tweezer and electrodes (3 mm diameter, catalog #450204, Harvard Apparatus), and 5 square wave electric pulses (45 V, 50 ms at 1 Hz) were delivered using an electroporator (ECM830, catalog #450662, Harvard Apparatus). The uterine horn was carefully placed back into the abdominal cavity, and the skin and peritoneum were sutured. The brains were harvested at E17.5 for immunostaining experiments.

### RNA extraction and cDNA synthesis

E12.5 neocortical primordium-derived cDNA was used as template to synthesis *Rbbp4* ORF. The neocortical primordia were dissected from E12.5 embryos, and RNA was isolated and purified using NucleoSpin RNA kit (Macherey-Nagel, catalog #740955). cDNA synthesis was subsequently carried out with 3 μg of RNA using the SuperScript IV Reverse Transcriptase kit (Invitrogen, catalog #18090010).

### DNA constructs

#### Rbbp4 *and* Cdon *knockdown constructs*

CRISPR/Cas 9-mediated genome editing was adopted to generate knockdown of respective genes in the neocortical progenitors. The guide RNA sequences were used from previously published sequences reported to have higher efficiency and less off-target effects (Joung et al., 2017). The single guide RNAs for *Rbbp4* [gRNA1-AACCCCGATTTGCGTCTCCG (Exon 5), gRNA2-TTGACGACGCAGTGGAAGAA (Exon 2)] were ligated into the BbsI digest of the pX458-Dual-Guide-Donor-Cas9-H2B-mCherry vector (Addgene plasmid, catalog #175570; RRID: Addgene_175570). The single guide RNAs [gRNA1-CTGTTACTGCCCGAATCTCA (Exon 3), gRNA2-TTGATGAAT CGAAATCACCC (Exon 4)] of *Cdon* gene were inserted into the BbsI digest of the pX458-Ef1a-Cas9-H2B-GFP vector (Addgene plasmid, catalog #159654; RRID: Addgene_159654).

#### *Rbbp4*-*GFP*

The vector *pCAG-IRES-EGFP* encoding IRES site along with an EGFP reporter cassette was a gift from Prof. Gordon Fishell, Harvard Medical School. The primers for mouse *Rbbp4* gene were designed such that they had an overlap with the vector sequence including the regions of restriction site EcoR1 and SwaI. The forward primer also contained a FLAG-tag sequence (GACTACAAAGACGATGACGACAAG, in bold letters) inserted between the ATG and the start of the gene as shown in Table 2. Using these specific primers, the *Rbbp4* ORF was amplified from the E12.5 neocortical primordium-derived cDNA. Platinum SuperFi II Green PCR Master Mix (Invitrogen, catalog #12369050) kit was used to amplify the *Rbbp4* ORF.

Primer sequences for amplifying *RBBP4* gene for the RBBP4-GFP construct. The forward primer contained a FLAG-tag sequence (marked in bold letters) inserted between the ATG and the start of the gene. The amplified *Rbbp4* ORF were ligated into the linearized vector pCAG-IRES-EGFP digested at the EcoR1 and SwaI sites. The presence of the insert in the vector was confirmed by Sanger sequencing.

#### *pEF1α-*Cdon

The vector pEF1α-*Cdon* was custom made by Twist Bioscience (https://www.twistbioscience.com). The vector encodes *Cdon* under the EF1α promoter, and the *Cdon* ORF is inserted at the EcoR1 and EcoRV sites of pEF1α vector (Twist expression vector). The presence of the insert in the vector was confirmed by sequencing.

### Cell culture and transfection

Neuro2A cells were used to estimate the extent of Rbbp4 knockdown in CRISPR/Cas 9-mediated genome editing and to validate the extent of overexpression of RBBP4 using pCAG-*Rbbp4*-IRES2-EGFP. Neuro2A cell line was obtained from ATCC (Sigma, catalog #89121404–1VL/ATCC; CCL-131) and cultured with DMEM (Invitrogen, catalog #11960069) supplemented with 20% fetal bovine serum (FBS; HiMedia, catalog #RM10434) and 1% Pen-Strep (Invitrogen, catalog #15140122) and 1% GlutaMAX (Invitrogen, catalog #35050061) in a humidified 5.0% CO2 incubator at 37°C. Neuro2A cells were seeded at a density of 1.5 × 10^5^ cells per well in a six-well plate. After 24 h of seeding the cells, transfection was performed with the RBBP4 gRNA1 and 2 constructs (1:1 ratio), pCAG-*Rbbp4*-IRES2-EGFP construct using the Lipofectamine 3000 Transfection Reagent (Invitrogen, catalog #L3000001), according to the manufacturer’s protocol. After 72 h of transfection, the cells were harvested using cold PBS, and the protein was extracted for SDS-PAGE and Western blotting experiment.

### SDS-PAGE and Western blotting

The cultured Neuro2A cells were harvested using cold PBS and homogenized in 1× RIPA buffer (50 Mm Tris-HCl, pH 8.0, 150 Mm NaCl, 1% NP40, 0.5% sodium deoxy cholate, 0.1% SDS), containing protease inhibitor cocktail (Sigma-Aldrich, catalog #P8340; Dhanya and Hasan, 2021). The homogenized cells were centrifuged at 12,000 rpm for 45 min at 4°C, and the supernatant was used as the total cell lysate. Protein quantity of the cell lysate was estimated by the bicinchoninic acid (BCA) assay (catalog #B9643). Laemmli sample buffer [5×; 10% SDS (w/v), 5% 2β-mercapto-ethanol, 50% glycerol, 0.02% bromophenol blue in 0.25 mM Tris-HCl] was added to the protein lysates and heated at 90°C for 5 min before loading onto SDS-PAGE (10% resolving gel and 5% stacking gel). The sample was run at 90 V in stacking gel and at 120 V in resolving gel using electrophoresis buffer (Tris 30.3 g, glycine 144.4 g, 10 g SDS in 1,000 ml of distilled water). Proteins were transferred onto the PVDF membrane using 1× transfer buffer (3.0285 g Tris, 14.2633 g glycine, 200 ml methanol in 800 ml of distilled water) at 90 V for 2 h. Further, the membranes were blocked with 5% Blotto (ChemCruz, catalog #sc-2325) for 1 h at 37°C and incubated with primary antibody (1:4,000 rabbit anti-RBBP4, catalog #A1490; RRID:AB_2761780, 1:2,000 mouse anti-PCNA, catalog #ab29; RRID:AB_303394 in 3% Blotto) for overnight on a platform shaker at 0–4°C. After washing with 1× TBST buffer thrice 10 min each on a platform shaker, blots were then incubated with secondary antibody for 2 h on a platform shaker. The secondary antibodies used were anti-mouse HRP (1:5,000; Sigma, catalog #A4416; RRID:AB_258167) and anti-rabbit HRP (1:5,000; Abcam, catalog #ab205718; RRID:AB_2819160). Bands of the blots were visualized with a chemiluminescence detection kit (ECL, Sigma-Aldrich, catalog #GERPN2209) and captured using a chemiluminescent detection system (ImageQuant LAS 4000, GE Healthcare) with Image Quant software.

### Isolation of neocortical primordia and nucleofection

E12.5 timed pregnant CD1-2019 dam was killed by cervical dislocation, the embryos were dissected out from the uterus, and the brains were removed in sterile cold L15 medium. As previously described in Muralidharan et al. (2017b), the neocortical primordium was dissected out and trypsinized with 0.25% trypsin solution at 37°C for 60–90 s. Equal volume of trypsin inhibitor solution (140 μl of 10 mg/ml soybean trypsin inhibitor, 100 μl of 1 mg/ml of DNase I in 10 mg/ml of HBSS) was added to neutralize trypsin. The cells were resuspended in 500 μl of complete neurobasal media containing B-27 supplement, GlutaMAX, and Penicillin-Streptomycin and counted using a hemocytometer. Approximately 2.5 × 10^5^ cells were nucleofected in 20 μl of 4D nucleofector solution (P3 Primary Cell 4D-Nucleofector Kit S; catalog #V4XP-3032) along with 500 ng of plasmid DNA following manufacturer’s protocol. After nucleofection, the cell suspension was seeded onto poly-D-lysine coated 8-well chamber slides (Nunc Lab Tek) and supplemented with 160 μl of complete neurobasal media. The cells were maintained in the CO_2_ incubator for 5 d and the old medium was replaced every day.

### Immunocytochemistry

Cultured cells were washed with 1× PBS thrice and fixed using 4% PFA in 1× PBS for 10 min on a platform shaker. After discarding the PFA, a quenching solution (375 μl of 2 M ammonium chloride, 100 μl of 2 M glycine in 10 ml of PBS) was added to the cells and incubated for 10 min. The cells were washed twice with PBS for 5 min each. The cells were then blocked for 1 h at 4°C in 0.1% Triton X-100 and 10% fetal bovine serum (Dhanya and Hasan, 2021) and stained overnight at 4°C with primary antibodies, including rabbit anti-RFP (1:500; Rockland Immunochemicals, catalog #600401379; RRID: AB_11182807), mouse anti-TUJ1 (1:1,000; Bio-Techne, catalog #MAB1195; RRID:AB_357520), rat anti-CTIP2 (1:1,000; Abcam, catalog #ab18465; RRID:AB_2064130), mouse anti-TLE4 (1:500; Santa Cruz Biotechnology, catalog #sc-365406; RRID:AB_10841582), and biotinylated goat anti-GFP (1:1,000; Abcam, catalog #ab6658; RRID: AB_305631), rabbit anti-RBBP4 (1:500; Abcam, catalog #AB 38135; RRID:AB_882293), mouse anti-RFP (1:500; Rockland, catalog #200-301-379, RRID:AB_2611063). The cells were washed thrice with PBS-T (0.1% Triton X-100 in 1× PBS) and incubated with suitable secondary antibodies for 2 h at room temperature, including streptavidin Alexa Fluor 488 (1:500; Invitrogen, catalog #S32354; RRID:AB_2315383), Goat anti-Rat Alexa Fluor 568 (1:500; Molecular Probes, catalog #A11077; RRID:AB_2534121), Goat anti-Mouse Alexa Fluor 568 (1:500; Molecular Probes, catalog #A11004; RRID:AB_2534072), Goat anti-Rabbit Alexa Fluor 568 (1:500; Molecular Probes, catalog #A11036; RRID: AB_10563566), Goat anti-Rabbit Alexa Fluor 488 (1:500; Molecular Probes, catalog #A11034; RRID:AB_2576217), and Donkey anti-Rabbit Alexa Fluor 647 (1:500; Molecular Probes, catalog #A32795; RRID:AB_2762835). Cells were washed thrice with PBS-T and incubated with DAPI (2.5 μg/ml) for 10 min. Cells were further washed thrice with 1× PBS and mounted in Fluoroshield medium (Sigma, catalog #F6182). Cells were imaged using an Olympus FV3000 confocal microscope with FV31S-SW 2.1 viewer software.

### Immunohistochemistry

The electroporated brains were fixed in 4% PFA in 120 mM phosphate buffer, pH 7.4, for 4–5 h at 4°C, transferred to 10, 20, and 30% sucrose sequentially. Brains were embedded in the SLEE cryo glue (Catalog #3000110, Micros Instruments) and cut into 20 μm sections. Antigen retrieval was performed by boiling the sections at 70°C in 10 mM citrate buffer, pH 6.0, followed by a wash with phosphate buffer, quenching for 30 min in 0.1 M glycine and blocking for 1 h in blocking solution containing 10% serum with 0.3% Triton X-100 at room temperature. Primary antibodies were incubated overnight at 4°C in blocking solution containing 5% serum with 0.3% Triton X-100. The following antibodies were used: rabbit anti-RBBP4 (1:500; Abcam, catalog #AB 38135; RRID: AB_882293), biotinylated goat anti-GFP (1:1,000; Abcam, catalog #ab6658; RRID: AB_305631), mouse anti-RFP (1:500; Rockland, catalog #200-301-379, RRID: AB_2611063). Subsequently, sections were washed once with PBS containing 0.1% Triton X-100 and twice in PBS, incubated with respective Alexa Flour secondary antibodies (1:1,000) in 5% blocking solution for 2 h at room temperature, and DAPI (2.5 μg/ml) for 10 min, washed again three times in PBS before mounting on microscopy slides with Fluorshield (Sigma, catalog #F6182). Images were acquired using Olympus FV300 confocal microscope with FV31S-SW 2.1 viewer software.

### Confocal imaging and image analysis

Confocal images were obtained using an Olympus FV3000 confocal microscope equipped with FV31S-SW 2.1 viewer software at 60× objective (PlanApoN, NA 1.42; Olympus oil-immersion). Images were acquired at 3.0 μm thickness intervals with frame size of 1,025 × 1,025 pixels. The images were merged, projected, and analyzed, using ImageJ (v.1.52p) software from the National Institutes of Health. Cell Counter plugin of the ImageJ software is used to count cells stained with various markers in the culture slides.

### Edu cell proliferation assay

The thymidine analog 5-ethynyl-2′-deoxyuridine (EdU) was used for the detection of proliferating cells in the mouse neocortical progenitor culture. EdU staining was performed using the Click-iT Plus EdU imaging kit with Alexa Fluor 555 picolyl azide (catalog #C10638) following the manufacturer’s instructions. After 3 h of nucleofection, progenitor culture was incubated with 10 μM EdU for 8 h. Following fixation with 4% PFA, cells were washed twice with 3% BSA in PBS for 5 min, then incubated with 0.5% Triton X-100 for 20 min at room temperature, and washed twice with 3% BSA in PBS for 5 min each. Subsequently, cells were incubated with Click-iT Plus reaction cocktail solution containing 1× Click-iT reaction buffer, copper protectant, Alexa Fluor 555 picolyl azide, and 1× reaction buffer additive for 30 min at room temperature followed by primary antibodies, including rabbit anti-RFP (1:500; Rockland Immunochemicals, catalog #600401379; RRID:AB_11182807), anti-PAX6 (1:500; BioLegend, catalog #901302; RRID:AB_2749901), and rabbit caspase-3 (1:500, Cell Signaling Technology, catalog #9662; RRID:AB_331439) overnight at 4°C. The next procedures were performed as mentioned in above, Immunocytochemistry section.

### Chromatin immunoprecipitation (ChIP)

The neocortical tissue from E12.5 embryos was isolated in cold 0.5% glucose in PBS containing 1× protease inhibitor mixture (Sigma-Aldrich, catalog #P8340). The isolated tissue was cross-linked immediately using 2 mM disuccinimidyl glutarate (ProteoChem, catalog #C1104) for 45 min and 1% formaldehyde (Invitrogen, catalog #28906) for 8 min, followed by quenching with 125 mM glycine. Cells were lysed and chromatin was sheared using the ultrasonicator (Covaris, catalog #S220) for 18 cycles of 60 s ON and 30 s OFF (5% duty cycle, 2 intensity, and 200 cycles per burst) to obtain fragment size of 100–300 bps (Muralidharan et al., 2018). Immunoprecipitation was performed using 4 μg antibody and 50 μg sheared chromatin. Antibodies used for ChIP were Rabbit RBBP4 (ABclonal, catalog #A1490; RRID: AB_2761780) and goat IgG (Genezyme Solutions, catalog #610680051730). The protein–DNA complex was pulled down using the Protein A-G magnetic beads (Dynabeads, Invitrogen) followed by washing thrice with low salt buffer (20 mM Tris-HCl, pH 8.0, 150 mM NaCl, 2 mM EDTA, 0.1% SDS, 1% Triton X-100), twice with high salt buffer (20 mM Tris-HCl, pH 8.0, 200 mM NaCl, 2 mM EDTA, 0.1% SDS, 1% Triton X-100), once with LiCl buffer (0.25 M LiCl, 1 mM EDTA, 10 mM Tris-HCl, pH 8.0, 1% NP-40, 1% sodium deoxycholate), and twice with TE buffer (10 mM Tris-HCl, pH 8.0, 1 mM EDTA). Chromatin was eluted by incubating the beads at 65°C for 30 min at 1,000 rpm in 300 μl of elution buffer (0.1 M NaHCO_3_, 1% SDS). The eluted chromatin was reverse cross-linked using sodium chloride, and the DNA was purified using phenol-chloroform-isoamyl alcohol (Invitrogen, catalog #15593-031) and then precipitated with ethanol and GlycoBlue (Invitrogen, catalog #AM9516). Precipitated DNA was resuspended in nuclease-free water (Invitrogen, catalog #AM9932) and quantified using Qubit 4 fluorometer (Thermo Fisher Scientific).

### Library preparation, sequencing, and ChIP-seq data analysis

Libraries were prepared using NEBNext Ultra II DNA Library Prep with Sample Purification Beads (E7103L), and sequencing was performed on an Illumina Hiseq 2500 platform using single end and 50 bp read protocol (Next-Generation Sequencing, NCBS). Two biological replicates were performed consisting of DNA isolated from the control neocortical primordia of E12.5 embryos. Approximately 33 million single-end reads were obtained per sample with a uniform distribution of reads across samples. FastQC v0.11.7 was performed as described previously (Andrews, 2010). First few bases having low quality was removed using Cutadapt v1.18 (Martin, 2011); parameters -*u* 5 and reads >=38 Phred scores were aligned to mouse reference genome (version GRCm38) genome using Burrows-Wheeler Alignment tool (BWA v0.7.17; Li and Durbin, 2009) using the default parameters. Aligned reads files were then interconverted and manipulated using Samtools (v1.6; Li et al., 2009) and BedTools (v2.25.0; Quinlan, 2014). Peaks were called with MACS2 v2.1.1.20160309 (Y. Zhang et al., 2008) using default parameters. Overlaps between ChIP-seq peaks were calculated using BEDTools intersect. The distribution of peaks (as intronic, intergenic, exonic, etc.) was annotated using HOMER (Unterwald et al., 2013; Heinz et al., 2010; Duttke et al., 2019). BigWig files, Metagene plots, and heatmaps of ChIP-seq were obtained with deepTools v3.1.3 (Ramírez et al., 2014). In all the downstream analysis input was subtracted from the respective samples reads using bamCompare function of deepTools. Venn, pie chart, and stacked bar plot were prepared using GraphPad Prism v9.4.0. Gene associations, GO terms, and KEGG pathways for RBBP4-bound elements were generated using G: Profiler script in R package (Raudvere et al., 2019). Motif identification on the genomic region bound by RBBP4 was performed using online MEME-ChIP (Machanick and Bailey, 2011) with the default parameters.

The identified motifs were compared with known motifs using TomTom (Gupta et al., 2007), and Find Individual Motif Occurrences (FIMO) was used to scan for motif-binding sites (Grant et al., 2011). The Integrative Genomics Viewer (IGV) browser was used to visualize the tracks (Robinson et al., 2009).

### RNA extraction and cDNA synthesis by reverse transcription

RNA was isolated from the cultured Neuro2A cells using the NucleoSpin RNA kit (Macherey-Nagel, catalog #740955) following the manufacturer’s protocol. The cultured Neuro2A cells after 48 h of transfection were harvested and homogenized in lysis buffer and the RNA was isolated by passing through the NucleoSpin gDNA removal column.

For cDNA synthesis, SuperScript IV Reverse Transcriptase kit (Invitrogen, catalog #18090010) was used. In total, 500 ng of total isolated RNA was used for each sample for cDNA synthesis following manufacturer’s protocol.

### Real-time quantitative PCR

Real-time quantitative PCRs (qPCRs) were performed using the SYBR Green PCR kit and 5 ng cDNA per sample (Thermo Fisher Scientific, catalog #4309155) on the QuantStudio 5 real-time PCR systems (Applied Biosystems). qPCR reaction was performed in duplicates. *Gapdh* was used as the house keeping gene to normalize the expression levels of genes of interest. Primers (sequences shown in Table 3) were designed by using Primer 3 (http://bioinfo.ut.ee/primer3-0.4.0/). The fold change of the gene expression of *Cdon* KD relative to the wild type was normalized according to the 2^−ΔΔCt^ method where ΔΔCt = [Ct (target gene) − Ct (*Gapdh*) *Cdon* KD − (Ct (target gene) −Ct (*Gapdh*) Control].

### Experimental design and statistical analysis

Mouse embryos of both sexes were used for the experiments. The statistical methods used in each experiment are described in the figure legends. The normality of the datasets was checked using the Shapiro–Wilk test. Statistical analysis was performed using Origin 8.0 software, GraphPad Prism v9.4.0 or R package. All bar graphs show means and standard error of means, and differences were considered as significant for *p* < 0.05 (*) and highly significant for *p* < 0.01 (**) and *p* < 0.001 (***) as determined using the two-tailed Student’s *t* test, Mann–Whitney test (two tailed) or one-way ANOVA as described in the figure legends.

## Results

### RBBP4 regulates the generation of neocortical neuronal populations and CTIP2-expressing neurons in the neocortical progenitors

To examine the expression of *Rbbp4* in the embryonic brain, we performed in situ hybridization on E12.5 brains (Fig. 1A,B). E12.5 was chosen because in the VZ the neuroepithelial to radial glia transition is complete, and the aRGs of the neocortex begin to divide asymmetrically to generate the diverse neuronal cell types (Fishell and Kriegstein, 2003; Tuoc et al., 2014). *Rbbp4* was seen to be strongly expressed in the proliferative zone of the neocortical primordium (Fig. 1B, Extended Data Fig. 1-1A), thereby suggesting its potential role in the development of neocortex.

To elucidate the functional role of RBBP4, we dissected out E12.5 neocortical primordia containing the progenitors from intact E12.5 hemispheres as shown in the schematic (Fig. 1A). We used a CRISPR/Cas9-mediated genome editing approach to perform RBBP4 knockdown in the mouse neocortical progenitors. We used a combination of guide RNAs (gRNAs) previously published to have higher predicted on-target knock down efficiencies and minimum off-target activity (Joung et al., 2017) targeting the exon 2 and 5 of *Rbbp4*. To determine the extent of RBBP4 knockdown, mouse Neuro2A cells were transfected with gRNAs of *Rbbp4*. Western blot data showed a 73% reduction in protein levels in cells transfected with *Rbbp4* gRNAs as compared with control (Fig. 1C,D; full blot in Extended Data Fig. 1-1B). To assess the reduction of RBBP4 protein in neocortical progenitors, we performed knockdown of RBBP4 using gRNAs in in vitro culture conditions and in E13 embryonic brains through in utero electroporation (Fig. 1E,G). There is a significant reduction in the percentage of RBBP4-positive cells from 90% (Cas 9 control) to 35% (RBBP4 KD) in culture conditions and 86 to 33% in electroporated cells (Fig. 1E,H), thereby validating the efficacy of the gRNAs.

To investigate the function of RBBP4 in neocortical neurogenesis, we nucleofected isolated E12.5 neocortical progenitors and examined the effects RBBP4 loss on the neuronal population (Fig. 2). At E12.5, a major proportion of the neocortical progenitors generate neurons as evidenced by the large percentage (87%) of control RFP nucleofected cells being TUJ1-positive neurons. *Rbbp4* knockdown resulted in significant reduction in this number to 60% (Fig. 2A; white arrowheads show transfected cells which are RFP+ and TUJ1+, white arrows show transfected cells which are RFP+ and TUJ1−). Next, we wanted to ascertain the neuronal subtype identity of the neurons. As the neuronal wave proceeds in the neocortical primordium, the CTPNs are predominantly generated first from E11.5 onward followed by a switch to the generation of SCPNs, E12.5 onward (Greig et al., 2013; Lodato and Arlotta, 2015). We wanted to determine whether the generation of the deep layer neurons, namely, CTPNs and the SCPNs, are affected upon *Rbbp4* knockdown. We used CTIP2-positive neurons as a marker for SCPNs and TLE4-positive neurons as a marker for CTPNs (Hevner 2007; Galazo et al., 2023). We observed that *Rbbp4* knockdown specifically affected the generation of CTIP2-expressing neurons. Their numbers were decreased upon loss of RBBP4 in the neocortical progenitors from 73 to 53% (Fig. 2B). Whereas reducing *Rbbp4* in the neocortical cells modestly increased the populations of TLE4-expressing cells though the result was not statistically significant (control, 45%; KD, 53%; Fig. 2C).

The absence of an effect on TLE4-expressing neurons following RBBP4 knockdown in neocortical progenitors may be attributed to the timing of the nucleofection at E12.5. By this stage, most of the Layer VI neurons are already formed in the neocortex, and there may also be a delay before the knockdown effects manifest in the in vitro system.

To eliminate potential off-target effects from using a combination of two guide RNAs against RBBP4, we validated our experiments with a single guide RNA targeting exon 2 of RBBP4. We observed that this single gRNA approach also led to a reduction in TUJ1-positive cells (Extended Data Fig. 2-1A,B) and in CTIP2-expressing neurons (Extended Data Fig. 2-1C,D). These results suggest that RBBP4 knockdown affects the generation of CTIP2-positive SCPNs. These findings indicate that RBBP4 plays a crucial role in regulating neurogenesis affecting CTIP2-expressing neuron generation in the neocortical progenitors.

### RBBP4 regulates progenitor proliferation in the cultured neocortical progenitors

To elucidate RBBP4’s impact on neocortical progenitor proliferation, we employed EdU, a marker for cell proliferation, to tag proliferating progenitors (Bordiuk et al., 2014). After 3 h of nucleofection, E12.5 neocortical progenitors were exposed to 10 μM EdU for 8 h. This 8 h time point aligns with the approximate E12.5 neocortex cell cycle length (Takahashi et al., 1995). To further understand the fate of the progenitors upon loss of RBBP4 in the neocortical progenitors, we coimmunostained the transfected cells with PAX6 (Englund et al., 2005). Upon RBBP4 depletion, we observed that there is a significant reduction in the percentage of EdU-positive and PAX6-positive cycling progenitors from 48% in the Cas 9 control cells to 33% in the RBBP4 KD neocortical cells (Fig. 3A,B; white arrowheads depict transfected GFP+, EdU+, and PAX6+ cells).

Overall, these findings indicate RBBP4’s crucial role in regulating neocortical progenitor proliferation, potentially contributing to the observed decrease in neuronal and CTIP2-expressing neuronal populations due to RBBP4 loss.

To ascertain if neocortical progenitor survival relies on RBBP4, we immunostained cells with caspase-3, an apoptosis marker (Iwasa et al., 2023). RBBP4 knockdown did not alter the proportion of caspase-positive cells in neocortical progenitor cultures (28% in both control and RBBP4 KD; Fig. 3C,D; white arrowheads indicate transfected GFP-positive and Caspase-positive cells). This observation suggests that the decrease in neuronal numbers stems from diminished neocortical progenitor proliferation due to RBBP4 knockdown, rather than apoptosis.

### RBBP4 is not sufficient to regulate neuronal fate specification in the neocortical progenitors

RBBP4 exists in both PRC2 that maintains gene silencing mainly through the deposition of H3K27me3 at promoter regions (Tanay et al., 2007; Ku et al., 2008) and in several histone deacetylase complexes that deacetylase H3K27 causing transcriptional repression (Clayton et al., 2006; Kelly and Cowley, 2013). Since RBBP4 could modulate transcriptional states depending on the composition of the chromatin remodeling complex it is a part of, we determined if overexpression of *Rbbp4* was sufficient to drive neuronal fate specification in the developing neocortical progenitors. E12.5 mouse neocortical progenitors were nucleofected with the pCAG-IRES2-EGFP construct in which the *Rbbp4* ORF was cloned under the constitutive CAG promoter and the progenitors were allowed to differentiate 5DIV. To determine the extent of over-expression of RBBP4 protein, mouse Neuro2A cells were transfected with pCAG-*Rbbp4*-IRES2-EGFP construct and Western blot data shows 10-fold increase in RBBP4 protein levels (Fig. 4A,B; full blot in Extended Data Fig. 4-1).

We observed that upon overexpression of RBBP4, there was no significant change in the TUJ1-positive neuronal populations, 83% GFP and 84.5% RBBP4 overexpression (Fig. 4C) indicating that overexpression of RBBP4 does not have any influence on the neocortical neuronal population (Fig. 2). We also noticed that overexpressing RBBP4 does not have any effect on CTIP2-expressing neurons (Fig. 4D) and TLE4-expressing neurons (Fig. 4E). This suggests RBBP4 is not sufficient to alter neuronal identity in the neocortical progenitors, and perhaps this could be attributed to its molecular role as a histone-binding protein and facilitator of binding of other histone-modifying proteins to the genomic loci but not perhaps affecting gene transcription on its own.

### Identification of RBBP4 genome-wide occupancy in the developing neocortex by ChIP sequencing

To understand the molecular function of RBBP4, we performed genome-wide occupancy analysis by chromatin immunoprecipitation followed by high-throughput sequencing (ChIP-seq) of the dissected and pooled neocortical primordia from E12.5 mouse brains as previously done in Muralidharan et al. (2017a) (Fig. 5A). Genomic distribution of RBBP4 ChIP-seq peaks revealed more binding in the intronic (36%, 17,144 peaks), intergenic (27.5%, 1,288 genes) regions followed by promoter (21%, 9,924 peaks) and exonic (6.6%, 3,120 peaks) regions of genes (Fig. 5B).

Since RBBP4 is part of NuRD and PRC2 complex which are well known to affect the state of histone marks, we investigated if there is any correlation between the RBBP4 binding sites and shared enhancer, active and repressive histone marks, namely, H3K4me1, H3K4me3, H3K27ac, and H3K27me3 based on published data (Sun et al., 2015; Fig. 5B). Interestingly, distribution of RBBP4 binding sites in the intergenic and intronic genomic regions is very similar to the binding sites of histones marks (Fig. 5B).

We performed Motif Enrichment (MEME) analysis of RBBP4 ChIP-seq peaks and found that two motifs with low *p* values contained the core homeobox motif TAATTA (Fig. 5C, Extended Data Fig. 5-2, Extended Data Table 5-1). This is not surprising given that we have shown previously (Muralidharan, et al., 2017a) that RBBP4 interacts with homeobox transcription factor (TF) LHX2. RBBP4 ChIP-seq in the ventral telencephalon (Price et al., 2022) also revealed similar binding motif suggesting that RBBP4 interacts with homeobox containing TFs to perform its chromatin regulatory functions in the mouse dorsal and ventral telencephalon—two most critical regions involved in the generation of glutamatergic and GABAergic neurons, respectively. We analyzed individual consensus binding sites in the four distinct DNA regions: promoter, intergenic, intron, and exon. Interestingly, we noted that the binding motif varies across these regions, potentially indicating regulation by different transcription factors (TFs) for each binding site (Extended Data Fig. 5-2).

To decipher the basic functional pathways in which RBBP4-bound target genes are involved in, we performed the REACTOME pathway analysis. Our top hits were nervous system development, axon guidance, signal transduction, and membrane trafficking suggesting RBBP4’s downstream effector genes are involved in neuronal processes in the developing neocortical primordium (Fig. 5D, Extended Data Table 5-2). Given RBBP4’s distribution across a variety of genomic regions, including promoters, intergenic, intronic, and exonic regions (Fig. 5B), we proceeded to decode the enriched GO biological pathways associated with each of these loci. Pathway analysis for each region uncovered analogous pathway enrichment patterns (Extended Data Fig. 5-1).

### CDON is a novel RBBP4 binding target gene

By pulse labeling isochronic cohorts of neural cells in the E12/13 neocortical primordium and scRNA seq analysis, Telley et al. (2019) identified differentially expressed transcripts in the progenitors and neurons. We compared our RBBP4 binding targets with this dataset to identify the AP-specific cortical genes regulating neurogenesis. We found that 1,325 of our RBBP4-bound target genes were expressed in the mouse E12.5 NCp and 32% of these (418 genes of 1,325) were enriched in expression in the APs and are genes which could be potentially involved in the birth and differentiation of a neuron (Fig. 6A, Extended Data Table 6-1). To understand the biological relevance of these genes expressed in the AP and bound by RBBP4, we performed the Gene Ontology (GO) pathway analysis. Our analysis revealed the top 10 biological pathways to be pertaining to nervous system development including neurogenesis, neuron differentiation, generation of neurons, cell division, cell cycle, etc. (Fig. 6B, Extended Data Table 6-2). Next, we carefully examined the genes in the GO pathway—neuron differentiation for potential downstream targets of RBBP4 involved in cortical neuronal generation and differentiation.

We uncovered a gene regulatory network with several known cortical genes such as transcription factors namely Hes1, Id4, Insm1, and Gli3 which are all negative regulators of cell proliferation and play critical role in the timing of neurogenesis in the developing neocortical primordium (Extended Data Table 6-2; Nakamura et al., 2000; Bedford et al., 2005; Wang et al., 2011). Given putative risk variants in RBBP4 have been linked to autism spectrum disorder (ASD; Firth et al., 2009), we aimed to investigate the autism-associated genes within RBBP4-bound loci. We compared our dataset of apical progenitor-specific RBBP4 binding targets with data from the SFARI gene database (https://gene.sfari.org; Fig. 6C). We found that 31 AP-specific RBBP4-bound genes are linked to ASD (Fig. 6C, Extended Data Table 6-3). Several of these are crucial for progenitor proliferation, namely, Pax6 (Warren et al., 1999), Lhx2 (Chou and O’Leary, 2013; Hsu et al., 2015), Aspm (Fish et al., 2006), Auts2 (Fair et al., 2023; Shimaoka et al., 2024), etc. This finding suggests a potential association between RBBP4 mutations and autism.

From this set of genes linked to ASD, we chose Cdon which functions as coreceptor of Patched1 in the Shh signaling pathway (Tenzen et al., 2006). It is also candidate gene associated with cortical malformation defects such as holoprosencephaly (Bae et al., 2011; Lu et al., 2018). *Cdon* null embryos display cortical thinning and defective neuronal differentiation (W. Zhang et al., 2006) but the neural cell type-specific role of Cdon in regulating the timing of deep layer neurogenesis in the neocortical progenitors is not known. *Cdon* is highly expressed in the dorsal telencephalon with almost no expression detected in the ventral telencephalon in both mouse (Fig. 6D, Extended Data Fig. 6-1) and human (Memi et al., 2018), thereby suggesting it could have a region-specific role in neocortical development which we wished to study.

We analyzed and identified substantial fold enrichment linked to RBBP4 peaks in the vicinity of the CDON genomic locus and find the TAATTA binding motif in several of these peaks (Extended Data Table 6-4). Genome browser views of the *Cdon* genomic loci shows RBBP4 binding close to the TSS and putative enhancers 9.5 kb upstream to TSS (Fig. 6E; data from our ChIP-seq analysis and Sun et al., 2015). The RBBP4 binding regions (boxed) are enriched with the enhancer mark H3K4me1, active mark H3K27ac, and active mark H3K4me3 (near TSS) but not the repressive H3K27me3 mark, putatively suggesting that perhaps Cdon is in active state when RBBP4 is bound.

### Cdon regulates neurogenesis and CTIP2-expressing neurons in the neocortical progenitors

Since the specific spatiotemporal role of Cdon in neocortical neurogenesis is unknown, we sought to functionally validate its role by downregulating *Cdon* using gRNAs and Cas9 (detailed in Materials and Methods) in E12.5 neocortical progenitors. We used a combination of gRNAs targeting the exon 3 and 4 of *Cdon* (Joung et al., 2017). To determine the extent of CDON knockdown, mouse Neuro2A cells were transfected with *Cdon* gRNAs, and qPCR was performed 48 h post transfection. A significant reduction in *Cdon* mRNA levels was observed upon *Cdon* knockdown as compared with that of controls (Extended Data Fig. 7-1).

*Cdon* knockdown in the mouse neocortical progenitor culture resulted in reduced neurogenesis from basal levels of 85 to 43% (Fig. 7A). More specifically CTIP2-expressing neurons were reduced in numbers (70 to 28%) whereas TLE4-expressing neuronal numbers remained unaffected (Fig. 7B,C). We also conducted an experiment using a single guide RNA targeting the exon 3 of *Cdon* which resulted in a significant reduction of TUJ1-positive neurons (Extended Data Fig. 7-2A,B) and CTIP2-expressing neurons (Extended Data Fig. 7-2C,D). These results unveil a novel role for CDON in regulating the numbers of CTIP2-expressing neurons, and its function parallels that of RBBP4 in neocortical progenitors.

### Role of CDON in regulating cell proliferation in the neocortical progenitors

CDON is a coreceptor of Patched1 that is involved in Shh signaling, and ablation of Shh at E12.5 has been reported to result in prolonged cell cycle length of cortical progenitors leading to shrunken cortex (Komada et al., 2008). Moreover, CDON has been implicated in the proliferation and differentiation control to promote myogenesis and neurogenesis in vitro (Takaesu et al., 2006; W. Zhang et al., 2006). Reduction of neurogenesis and CTIP2-expressing neurons in the neocortical progenitors upon loss of function of CDON motivated us to further investigate the effect of CDON on cell proliferation. To address this, we performed EdU incorporation assay where the E12.5 neocortical progenitor culture 3 h post transfection was incubated with 10 μM EdU for a period of 8 h. To explore the impact of CDON loss on the fate of neocortical progenitors, we coimmunostained the transfected cells for PAX6. We found that reducing CDON in neocortical cells led to a significant decrease in EdU-positive and PAX6-positive cycling progenitors, dropping from 48% in controls to 21% in CDON loss of function condition (Fig. 8A,B; white arrowheads show transfected cells which are GFP-positive, EdU-positive, and PAX6-positive). Our data is in accordance with the previous findings where reduced proliferation was observed in the primary neural progenitor cultures from the E14.5 *Cdon* knock-out mice that displayed cortical thinning (W. Zhang et al., 2006). These results underscore CDON’s critical role in regulating the proliferation of neocortical progenitors, which could significantly impact the population of TUJ1- and CTIP2-expressing neurons.

To rule out the involvement of apoptosis, we immunostained the transfected neocortical progenitor culture with anti-Caspase 3 antibody (Fig. 8C,D; white arrowheads indicate transfected cells which are GFP-positive and Caspase-positive). We observed no significant difference between control and *Cdon* knockdown (Fig. 8C,D). Though previous studies have demonstrated the importance of CDON in regulating apoptosis in human cancer cells and cell line studies (Delloye-Bourgeois et al., 2013; Uluca et al., 2022), our data shows that reducing CDON in the neocortical progenitors do not have any immediate effect on its viability.

### Overexpression of CDON in RBBP4-deficient neocortical progenitors rescues the reduced neurogenesis phenotype

To further explore the functional link between RBBP4 and its binding target *Cdon*, we investigated whether overexpressing CDON could mitigate the effects observed from RBBP4 loss in neocortical progenitors (Fig. 9).

The pEF1α-Cdon expressing vector (CDON OE), which expresses CDON, was nucleofected along with Rbbp4 gRNAs (RBBP4 KD+CDON OE) in E12.5 neocortical progenitors and cultured for 5 d in vitro (Fig. 9). Overexpressing CDON in RBBP4 knockdown neocortical progenitors significantly restored CTIP2-expressing neurons from 53% in Rbbp4 KD cells to 79% Rbbp4 KD+CDON OE progenitors (Fig. 9A,B; white arrowheads in Fig. 9A indicate transfected cells that are RFP-positive and CTIP2-positive, while white arrows indicate transfected cells that are RFP-positive and CTIP2-negative). Additionally, overexpressing CDON in the RBBP4 KD neocortical progenitors also restored the neuronal population from 60% in RBBP4 KD condition to 81% in the Rbbp4 KD+CDON OE cells (Fig. 9C,D; white arrowheads in Fig. 9C indicate transfected cells that are RFP-positive and TUJ1-positive, white arrows indicate transfected cells that are RFP-positive and TUJ1-negative). In contrast, control GFP nucleofection along with RBBP4 gRNAs did not restore the number of neurons or CTIP2-positive neuronal numbers.

These results indicate that CDON overexpression can rescue the reduced neurogenesis and altered neuronal subtype specification observed in the RBBP4-deficient neocortical progenitor cultures. This experiment shows that CDON over-expression functionally compensates for the effects of RBBP4 loss, implying that CDON may function downstream of RBBP4 in the development of neocortical neurons.

## Discussion

Unique transcriptional programs establish spatiotemporal neuronal identity in the apical progenitor. The apical progenitor then begins to differentiate into basal progenitors expressing proliferative transcripts, then newly post miotic neurons expressing neurogenic transcripts, and finally mature neuron expressing neuronal transcripts. The neurons migrate radially into the cortical plate to occupy their spatially distinct destined positions in the neocortex (Angevine and Sidman, 1961; Nadarajah et al., 2003; Kriegstein and Noctor, 2004; Hatanaka et al., 2016). During neocortical development, chromatin regulation plays a crucial role by altering chromatin accessibility to ensure the expression of dynamic transcriptional states governing cell fate choices (Telley et al., 2019). Mutations in epigenetic modifiers and chromatin regulators lead to a range of neurodevelopmental disorders (Park et al., 2016; Stessman et al., 2017). Putative risk variants in RBBP4 have been associated with ASD and intellectual disability (Firth et al., 2009). Here we determine the role of WDR (WD40 Repeat) domain containing histone chaperone RBBP4 which is part of several chromatin-modifying complexes including NURD and the Polycomb complex in regulating corticogenesis (Kuzmichev et al., 2002; Feng and Zhang, 2003; Conway et al., 2018).

In this study, we have identified the novel role for RBBP4 in regulating neocortical progenitor proliferation and neurogenesis. Our data demonstrates the critical importance of RBBP4 in maintaining the proliferative state of neocortical progenitors during neurogenesis (Figs. 2, 3). EdU assays indicate that RBBP4 depletion in E12.5 neocortical progenitors significantly reduces the proportion of cycling progenitors, likely due to premature cell cycle exit and differentiation into neurons. This reduction in the progenitor pool eventually leads to fewer TUJ1+- and CTIP2-expressing neuronal populations, similar to what is observed in Pax6 and Lhx2 knock-out mutants (Warren et al., 1999; Chou and O’Leary, 2013; Hsu et al., 2015). Given that RBBP4 interacts with Lhx2, it appears similarly crucial for maintaining progenitor proliferation (Muralidharan et al., 2017a). Other NuRD complex components, such as HDAC1/2, CHD4, LSD1, and MBD3, also play significant roles in regulating progenitor proliferation (Hagelkruys et al., 2014, 2016; F. Zhang et al., 2014; Nitarska et al., 2016; Moon et al., 2017; D’Souza et al., 2021). Our findings emphasize RBBP4’s essential role in supporting neocortical progenitor proliferation, contributing to the body of evidence on how NuRD complex subunits regulate progenitor proliferation during corticogenesis. Moreover, our study adds to the understanding of RBBP4’s context-dependent mechanisms in progenitor maintenance. For instance, RBBP4 and RBBP7 depletion has been shown to block cell cycle progression in preimplantation mouse embryos, leading to dysregulation of cell cycle-related genes and those involved in transcription and lineage development (Xiao et al., 2022). Similarly work by Schultz et al. (2018) and Schultz-Rogers et al. (2022) on zebrafish brains found that RBBP4 loss resulted in neural progenitor apoptosis and an increased number of M-phase cells in the mutant midbrain, suggesting that RBBP4 is involved in cell cycle regulation and progenitor survival. The varied roles of RBBP4 may be attributed to its interacting partners in different cell types, which likely influence downstream effector genes and the observed effects on progenitor proliferation.

Our study highlights the functional role of RBBP4 in regulating deep layer neurogenesis, as demonstrated by our experiments with E12.5 progenitors. RBBP4 is abundantly expressed in the mouse neocortex at E12.5 and remains expressed at E15.5 (Klingler et al., 2021), suggesting its temporal role in neurogenesis during neocortical development. Further studies are needed to delineate the importance of RBBP4 in upper layer neuron regulation.

Overexpression of RBBP4 was not a sufficient condition for the process of neurogenesis (Fig. 4). RBBP4 alters DNA and histone topology (Verreault et al., 1996; Murzina et al., 2008), but it requires cooperation with other regulatory factors such as histone-modifying enzymes (e.g., HDAC, LSD1, EZH2) found in NuRD and PRC2 chromatin complexes to modulate gene expression at specific sites (Tanay et al., 2007; Ku et al., 2008; Muralidharan et al., 2017a; Mu et al., 2022). However, since histone-modifying enzymes participate in multiple complexes, they can be rate limiting. Therefore, RBBP4 overexpression alone is inadequate to alter neurogenesis. Another reason could be the necessity for post-translational modifications such as phosphorylation, acetylation, and methylation that can affect the activity or specificity of RBBP4. Furthermore, the interactions leading to these modifications could be limiting in the progenitor. Overall, multiple factors need to work together to regulate neurogenesis, and RBBP4 is just one of many regulatory proteins involved.

To explore the potential downstream effector mechanisms of RBBP4 function, we performed RBBP4 ChIP-seq on E12.5 neocortical primordium to determine the genome-wide occupancy profile. Through this, we identified several genes that play critical roles in neurogenesis, namely, *Hes1, Id4, Insm1*, and *Cdon*, which RBBP4 binds to (Figs. 5, 6). To validate the functional role of one of these genes, CDON—a coreceptor of Patched1 involved in Shh signaling—we performed *Cdon* knockdown experiments. Our findings revealed a reduction in neurogenesis, specifically affecting CTIP2-positive neurons (Fig. 7), which is consistent with the observed cortical thinning and reduced differentiation seen in *Cdon* null embryos (W. Zhang et al., 2006). CDON has been found to positively regulate Sonic hedgehog (Shh) signaling in specific regions of the developing embryo (W. Zhang et al., 2006). Shh plays a crucial role in patterning and cell fate specification as well as maintaining the stem cell niche in the telencephalon (Dahmane et al., 2001; Lai et al., 2003; Machold et al., 2003; Palma and Ruiz i Altaba, 2004; Palma et al., 2005). Since CDON is expressed in proliferating VZ progenitor cells, it is possible that a reduction in CDON could impact Shh signaling in these cells contributing to the observed reduction in neocortical progenitor proliferation. This decrease in progenitor proliferation may, in turn, affect the generation of CTIP2-expressing neurons (Figs. 7, 8).

Overall, our data demonstrates the essential role of CDON in regulating proliferation and neuronal specification in the neocortical progenitors. Additionally, previous studies have shown that ablation of Shh at E12.5 leads to prolonged cell cycle length of cortical progenitors, which results in a shrunken cortex (Komada et al., 2008). *Cdon* knockdown resulted in a phenotype similar to that of Rbbp4 knockdown, and CDON overexpression can rescue the reduction of neurogenesis observed with the loss of RBBP4 in the neocortical progenitors (Fig. 9). This suggests a functional link between RBBP4 and its target gene CDON.

Molecularly RBBP4 functions as a histone chaperone and helps reposition nucleosome cores in an ATP-dependent manner to enable enzymes of the chromatin-associated complexes to modify histones and remodel chromatin structure (Verreault et al., 1996; Murzina et al., 2008; Millard et al., 2016). Since RBBP4 is part of both the PRC2 and the NuRD complexes, its precise molecular role in regulating chromatin dynamics will be interesting to explore. Whether RBBP4 functions as a repressor or activator and in the context of which complex will reveal insights into the dynamic epigenetic regulation occurring within neocortical progenitors to generate the different layers of glutamatergic neurons.

In summary, we have characterized the novel functional cellular role of RBBP4 in neocortical development and identified its genome-wide binding occupancy profile. Our study demonstrates the critical role of RBBP4 in regulating progenitor proliferation and neuronal specification. We have identified *Cdon* as one of the novel binding targets of RBBP4. Based on our study, we show that CDON plays a novel role in regulating proliferation and deep layer neurogenesis in the neocortical progenitors (Fig. 10). Overexpression of CDON can rescue the effects of RBBP4 loss and restore neurogenesis, indicating a functional association between RBBP4 and CDON. These findings hold significant importance in the context of comprehending how disrupted chromatin regulation may affect cellular mechanisms in neurodevelopmental disorders, and they offer valuable insights into how potential disease risk variants in RBBP4 can influence cerebral cortical development in individuals with autism.

## Extended Data

**Figure 1-1:**
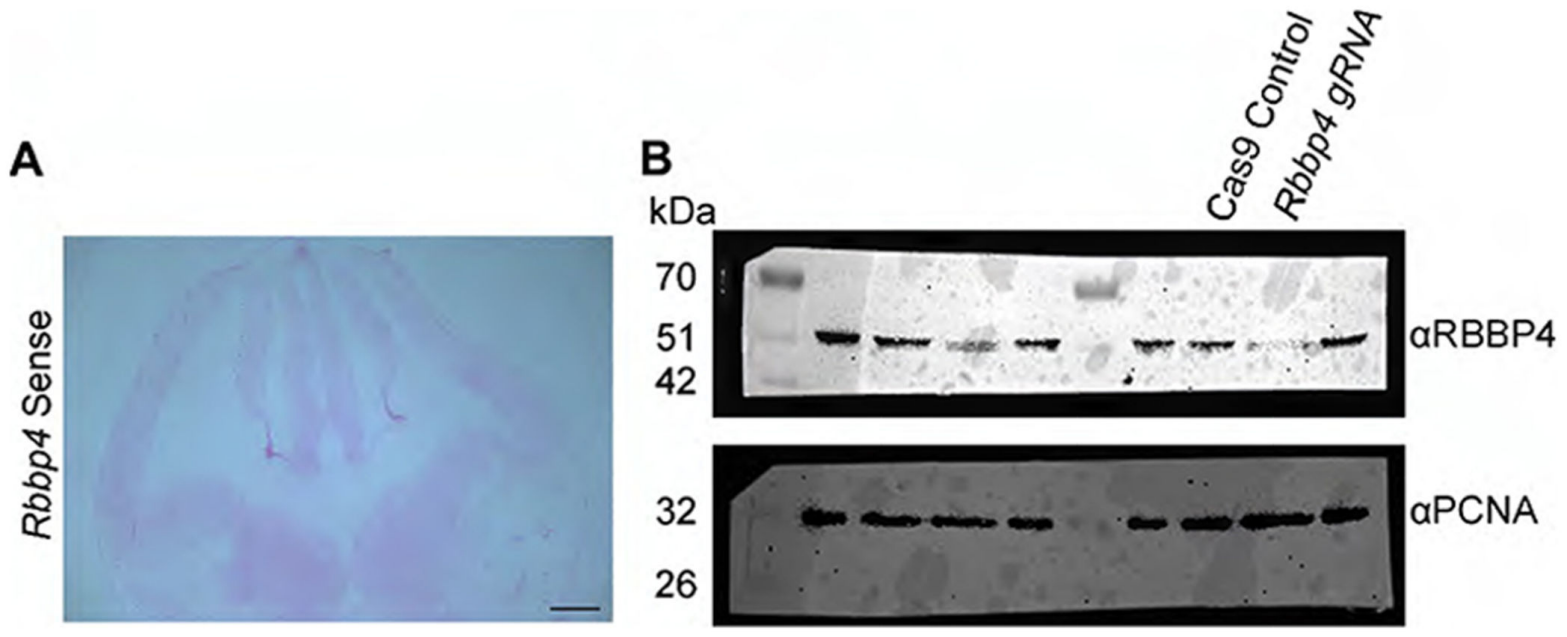
In Situ Hybridization (ISH) and western blot analysis of RBBP4 in the neocortical primordium (Related to Figure 1) (A) The In Situ Hybridization (ISH) image of the E12.5 mouse brain, probed using sense control probes against Rbbp4 mRNA, shows no non-specific binding of the probe to the tissue. Scale bar -1 mm. (B) Complete western blot of figure 1C showing reduced RBBP4 levels in the Neuro2A cells transfected with *Rbbp4* sgRNAs (right lane) as compared to the control cells (left lane).

**Figure 2-1:**
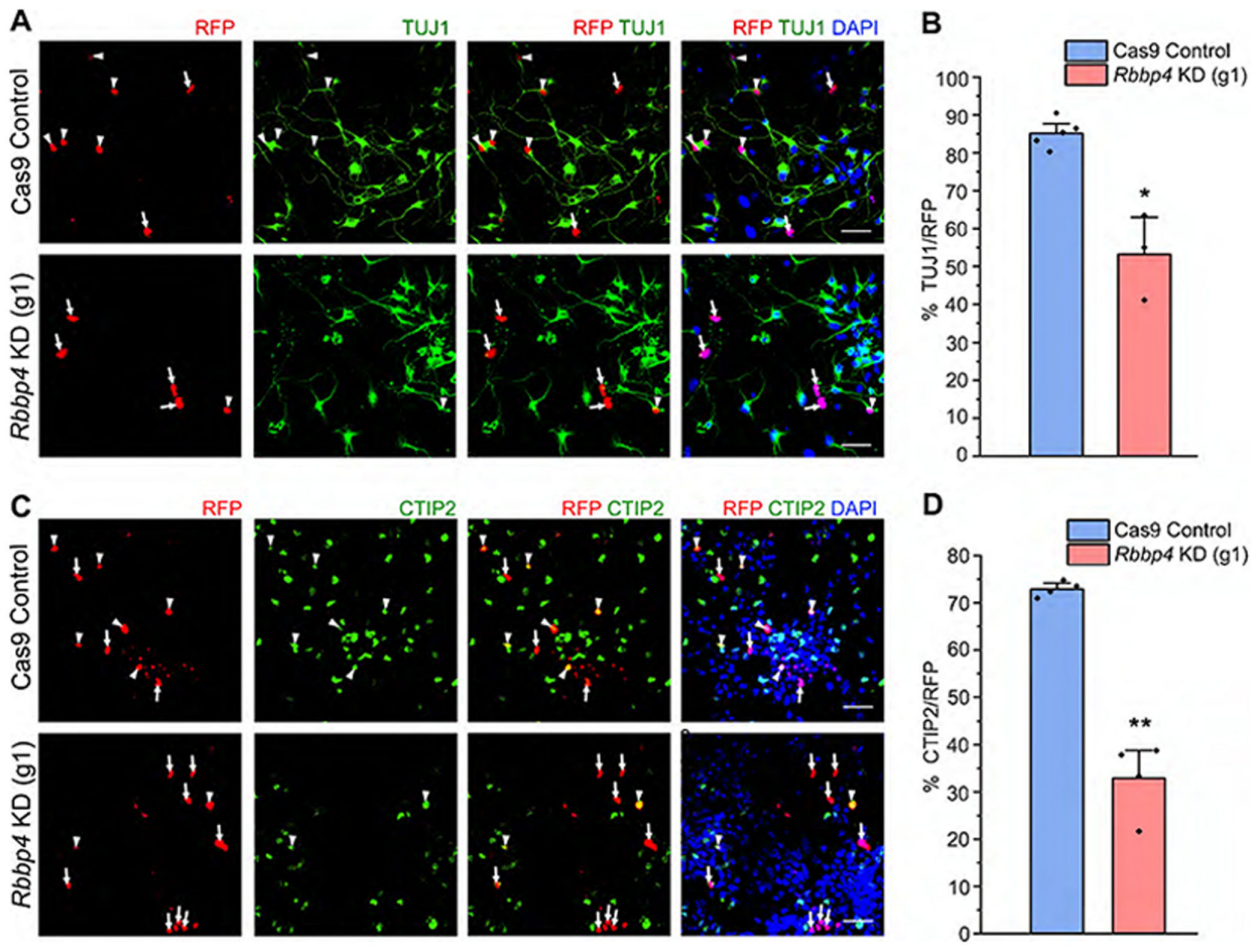
RBBP4 knockdown using a single gRNA against Rbbp4 results in a reduction of both total neurons and CTIP2-expressing neurons. (Related to Figure 2). Neocortical progenitors of E12.5 mouse embryo transfected with a single guide RNA (Cas9-mCherry-Rbbp4) targeting the exon 2 of *Rbbp4*. (A and C) Confocal images showing the cultured neocortical progenitors stained with anti-TUJ1 (A) and CTIP2 (C) antibodies after 5DIV (B and D) Bar graph with the percentage of TUJ1 + ve (B) and CTIP2 + ve (D) cells of the total RFP transfected cell populations for the Cas9 control and Rbbp4 KD neocortical progenitors. Data are presented as mean ± SEM; Two-tailed Student's t-test; * *p* < 0.05 and ** *p* < 0.01. White arrowheads indicate transfected cells which express RFP and the indicated markers of panels A and C, and white arrows show transfected cells which express RFP and do not express the indicated markers. Scale bars for (A) and (C) are 30μm.

**Figure 4-1:**
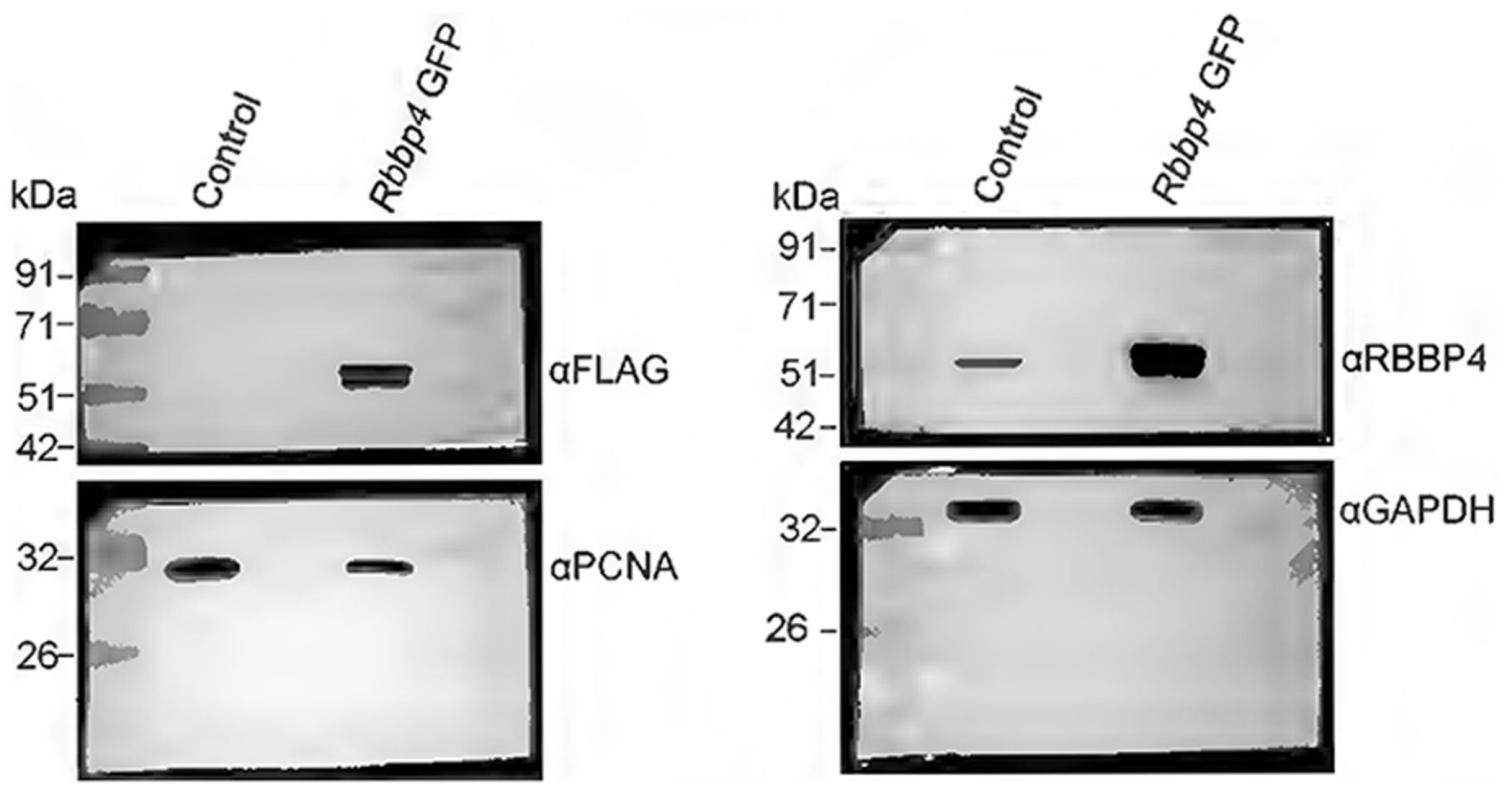
Western blot showing increased RBBP4 levels in the Neuro2A cells transfected with *Rbbp4*-GFP vector (Related to Figure 4A) Western blot showing increased expression of RBBP4 protein levels in the Neuro2A cells transfected with *Rbbp4*-GFP vector (*pCAG-IRES-EGFP*) as compared to the control vector (*pCAG-IRES-EGFP*). (Right Panel) Immunoblots probed with antibodies against FLAG (55 kDa) which is tagged to the RBBP4-ORF in the overexpression constructs and PCNA (28 kDa). (Right Panel) Immunoblots probed with antibodies against RBBP4 (55 kDa) and GAPDH (37 kDa).

**Figure 5-1:**
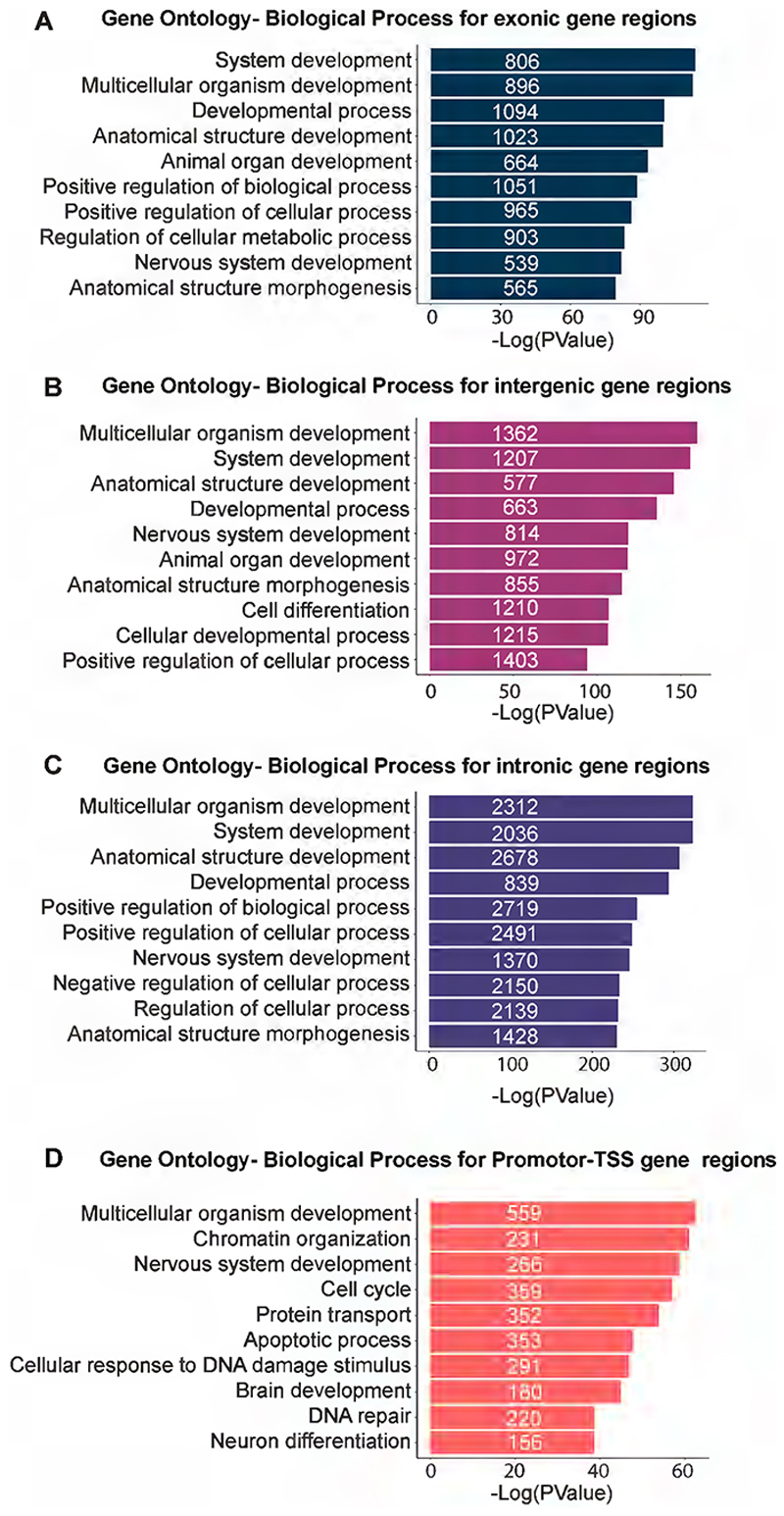
Gene Ontology analysis of the RBBP4 bound genes at various genomic loci (Related to Figure 5B) Gene Ontology analysis of the various enriched pathways of RBBP4 bound genes at (A) exonic (B) intergenic and (C) intronic regions (D) promoter-TSS genomic regions.

**Figure 5-2:**
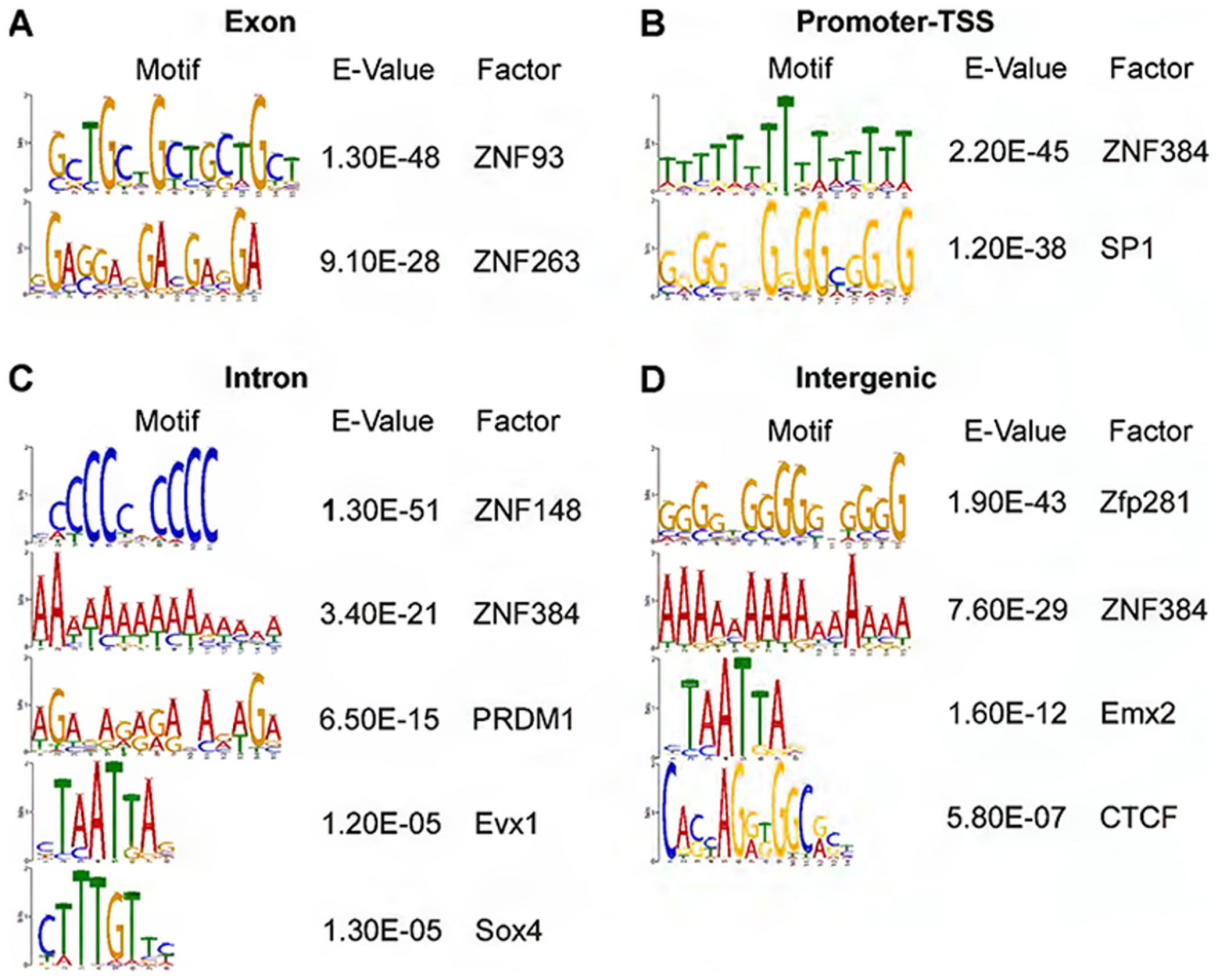
MEME plot showing the DNA motifs of different genomic loci identified in RBBP4 ChIP-seq peaks (Related to Figure 5C) MEME plot showing the most significantly enriched DNA motifs identified in RBBP4 ChIP-seq peaks for various genomic loci such as (A) Exon, (B) Promoter-TSS, (C) Intron and (D) Intergenic regions.

**Figure 6-1:**
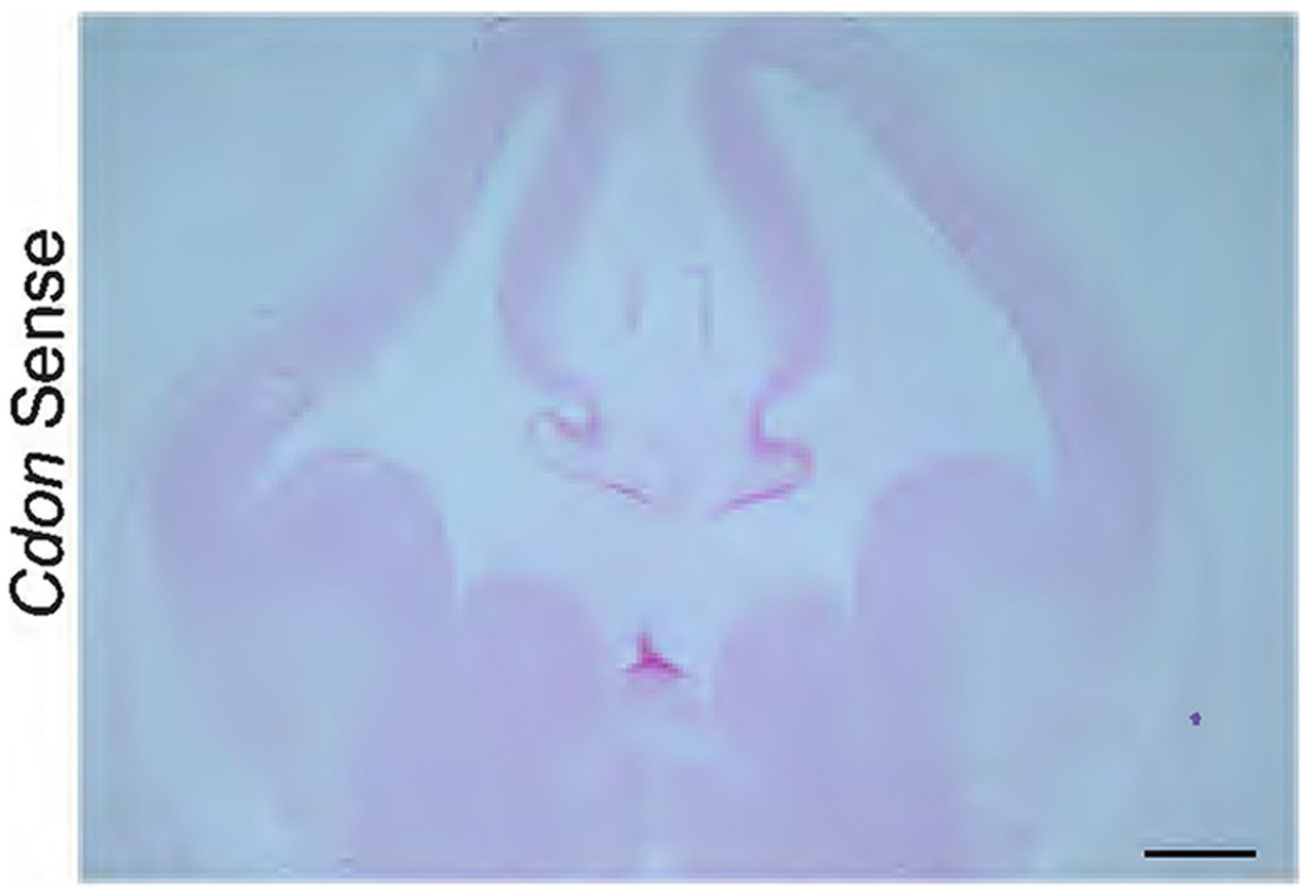
In Situ Hybridization (ISH) analysis for *CDON* in the neocortical primordium (Related to Figure 6D). The In Situ Hybridization (ISH) image of the E12.5 mouse brain, probed using sense control probes against *Cdon* mRNA, shows no non-specific binding of the probe to the tissue. Scale bar -1 mm.

**Figure 7-1:**
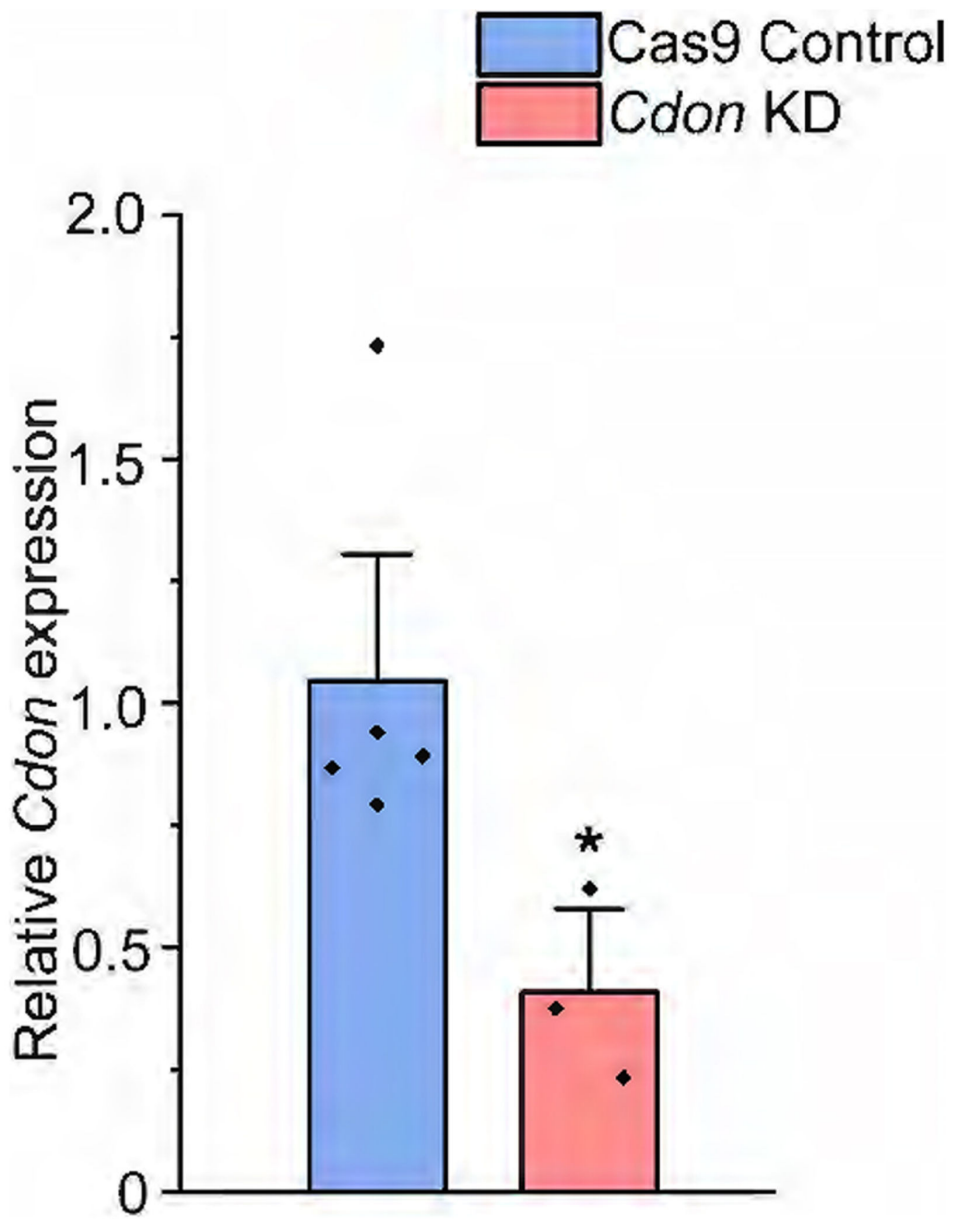
Fold Changes in *Cdon* expression in *RBBP4* KD and *Cdon* KD Neuro2A cells (Related to Figure 7) Bar graph indicate relative expression of *Cdon* mRNA levels in the *Cdon* KD Neuro2A cells. All measurements are done by qRT-PCR and *Gapdh* was used as the house keeping gene to normalise the expression levels of *Cdon*. Data are presented as mean ± SEM; Mann-Whitney test (Two tailed); * *p* < 0.05.

**Figure 7-2:**
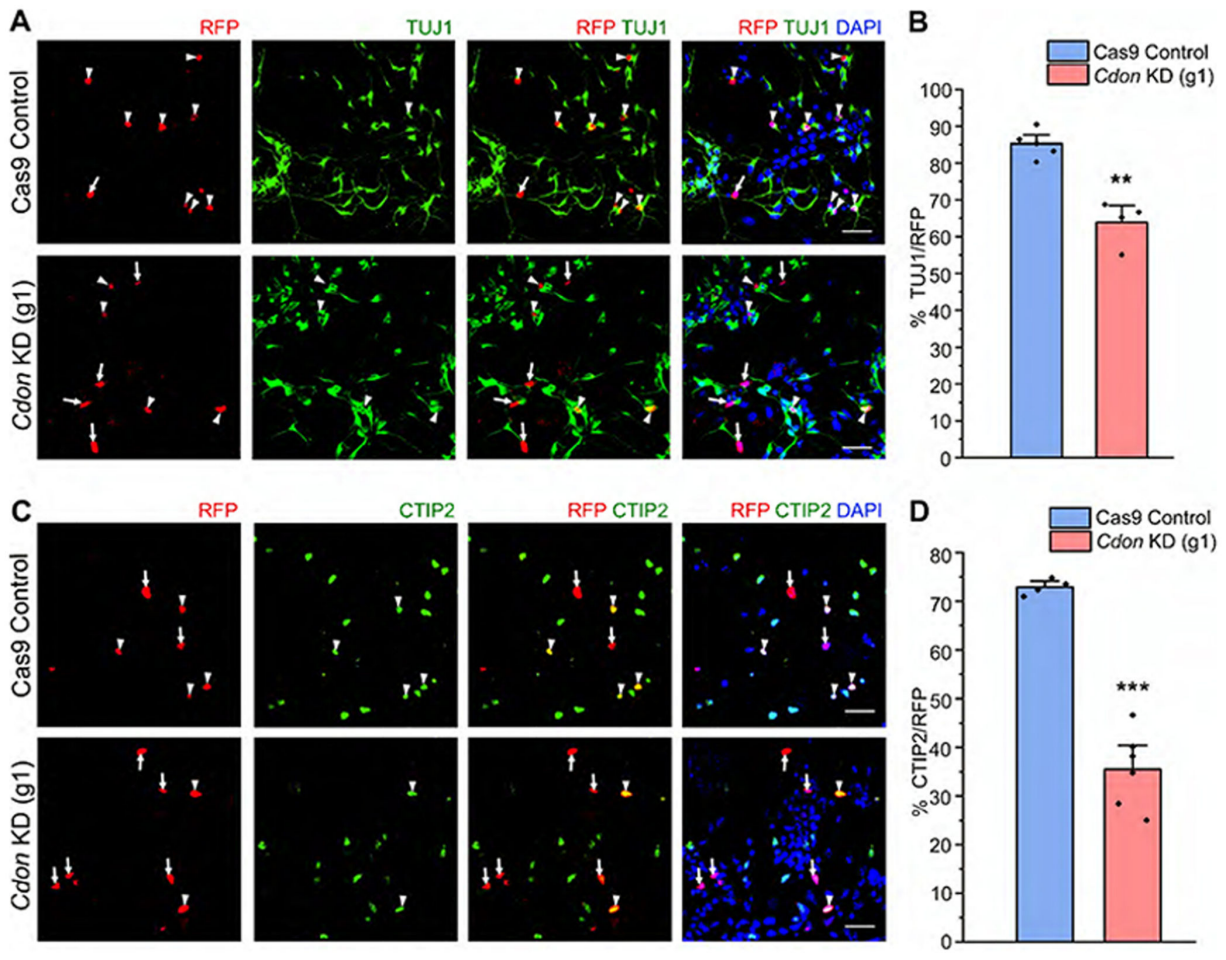
CDON knockdown using a single gRNA against Cdon results in a reduction of both total neurons and CTIP2-expressing neurons. (Related to Figure 7). Neocortical progenitors of E12.5 mouse embryo transfected with a single guide RNA (Cas9-*Cdon*) targeting the exon 3 of *Cdon*. (A and C) Confocal images showing the cultured neocortical progenitors stained with anti-TUJ1 (A) and CTIP2 (C) antibodies after 5DIV. (B and D) Bar graph with the percentage of TUJ1 + ve (B) and CTIP2 + ve (D) of the total RFP transfected cell populations for the Cas9 control and *Cdon* KD neocortical cells. Data are presented as mean ± SEM; Two-tailed Student's t-test; ** *p* < 0.01 and *** p < 0.001. White arrowheads indicate transfected cells which express RFP and the indicated markers of panels A and C, and white arrows show transfected cells which express RFP and do not express the indicated markers. Scale bars for (A) and (C) are 30μm.

## Supplementary Material

Supplementary Figure 1-1

Supplementary Figure 2-1

Supplementary Figure 4-1

Supplementary Figure 5-2

Supplementary Figure 5-1

Supplementary Figure 6-1

Supplementary Figure 7-1

Supplementary Figure 7-2

table-Table 5-1 Enriched DNA motifs identified in RBBP4 ChIP-seq peaks (Related to Figure 5C) Spreadsheet showing the summary of significantly enriched DNA motifs identified in RBBP4 ChIP-seq peaks.

table-Table 5-2 Gene Ontology analysis of the RBBP4 bound genes from ChIP-seq data (Related to Figure 5D) Spreadsheet showing the Gene Ontology analysis of the various enriched pathways of RBBP4 bound genes.

table-Table 6-1 RBBP4 bound genes shared among apical and basal progenitor neurons (Related to Figure 6A) Spreadsheet showing the list of RBBP4 bound genes and those shared among by the apical and basal progenitor and neurons (data from Telly et al., 2019).

table-Table 6-2 Gene Ontology analysis of the enriched biological pathways of RBBP4 in the apical progenitors (Related to Figure 6B) Spreadsheet showing the Gene Ontology analysis of the various enriched pathways of RBBP4 gene in the apical progenitors (data from Telley et. al 2019).

table-Table 6-3 Analysis of apical progenitor specific RBBP4-bound genes associated with autism spectrum disorder risk genes (Related to Figure 6C) Spreadsheet showing the autism associated risk genes within the apical progenitor specific RBBP4-bound loci [Our ChIP seq data compared with data from Safari gene database (https://gene.sfari.org)].

table-Table 6-4 Analysis of RBBP4 peaks associated with the Cdon genomic locus (Related to Figure 6E) Spreadsheet showing the various RBBP4 peaks associated with the Cdon genomic locus from ChIP-seq analysis.

## Figures and Tables

**Figure 1:**
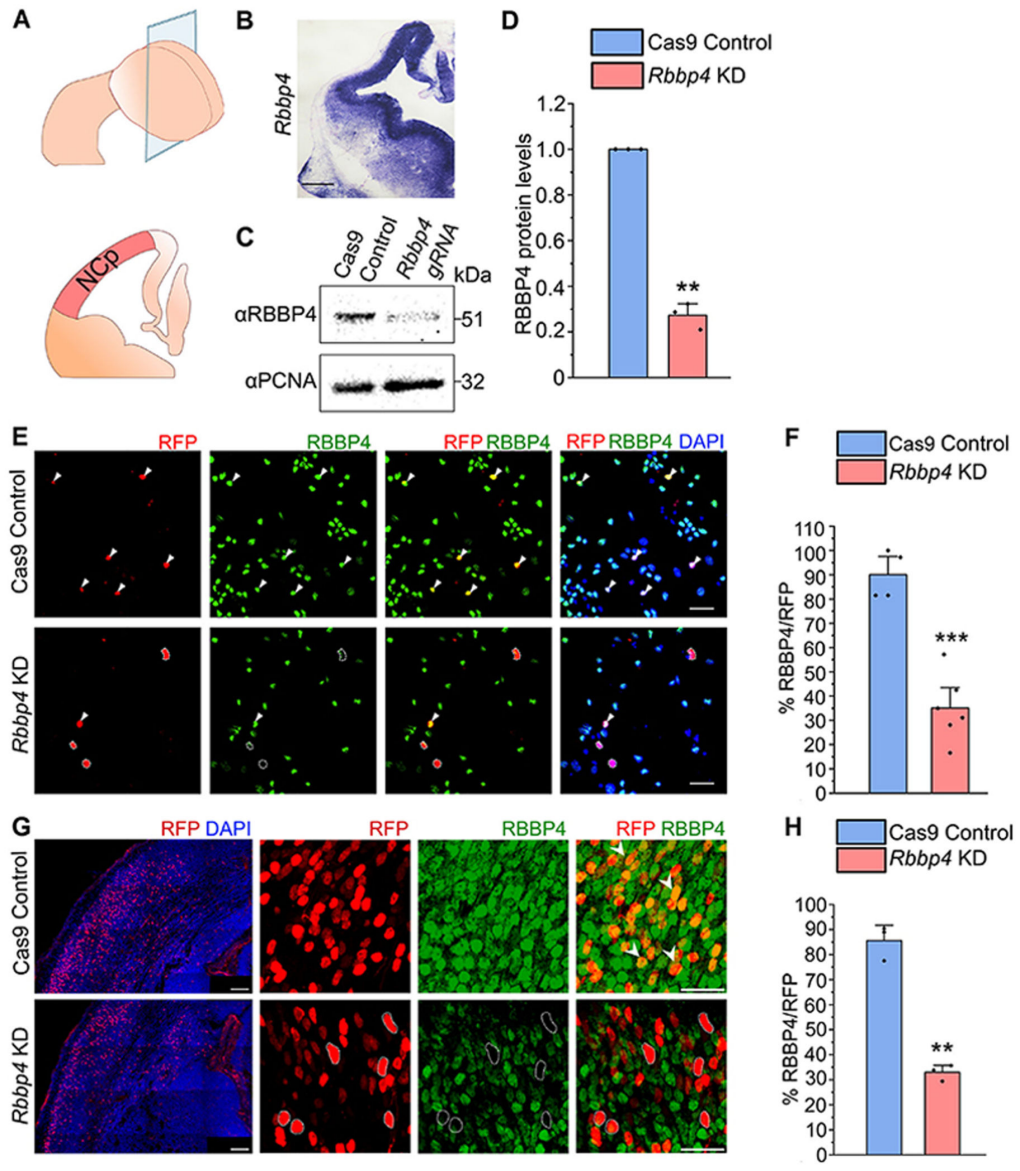
Loss of RBBP4 in the transfected cells of cultured neocortical progenitors. ***A***, Schematic diagram illustrating the neocortical primordium (NCp) used for dissection. ***B***, In situ hybridization (ISH) image showing the expression of *Rbbp4* (refer to Extend Data Fig. 1-1 for more details). Scale bar, 100 μm. ***C***, Western blot showing reduced RBBP4 levels in the Neuro2A cells transfected with *Rbbp4* sgRNAs (right lane) as compared with the Cas9 control cells (left lane; refer to Extend Data Fig. 1-1 for more details). ***D***, Bar graph showing the fold change in RBBP4 protein levels in the control and *Rbbp4* knockdown in Neuro2A cells. Fold changes were normalized to PCNA levels for each sample. ***E***, Confocal images showing the cultured neocortical cells stained with anti-RFP (red, left Panel) and anti-RBBP4 (green) antibodies after 5DIV. Scale bar, 30 μm. ***F***, Bar graph showing the percentage of RBBP4-positive cells of the RFP transfected cells for control and *Rbbp4* KD in neocortical progenitors. ***G***, Confocal images showing the mouse brain electroporated at E13 with control (Cas9-mCherry plasmid, top panel) and RBBP4 guide RNAs (gRNA1 and gRNA2, bottom panel) and harvested for immunostaining at E17.5. Left panel shows the region of electroporated cells in the neocortical primordium (RFP/DAPI). Electroporated brain sections were immunostained with anti-RFP (red) and anti-RBBP4 (green) antibodies at E17.5 and imaged at 40×. Scale bar for left panel is 100 μm and for other panels is 30 μm. ***H***, Bar graph showing the percentage of RBBP4-positivecells of the RFP transfected cells for control and *Rbbp4* KD cells in the electroporated mouse neocortex. Data are presented as mean ± SEM; two-tailed Student’s *t* test; **p < 0.01 and ***p < 0.001. White arrowheads in panels ***E*** and ***G*** indicate transfected cells which express RFP and RBBP4. White dotted circles indicate cells which are RFP-positive with loss of RBBP4 protein.

**Figure 2:**
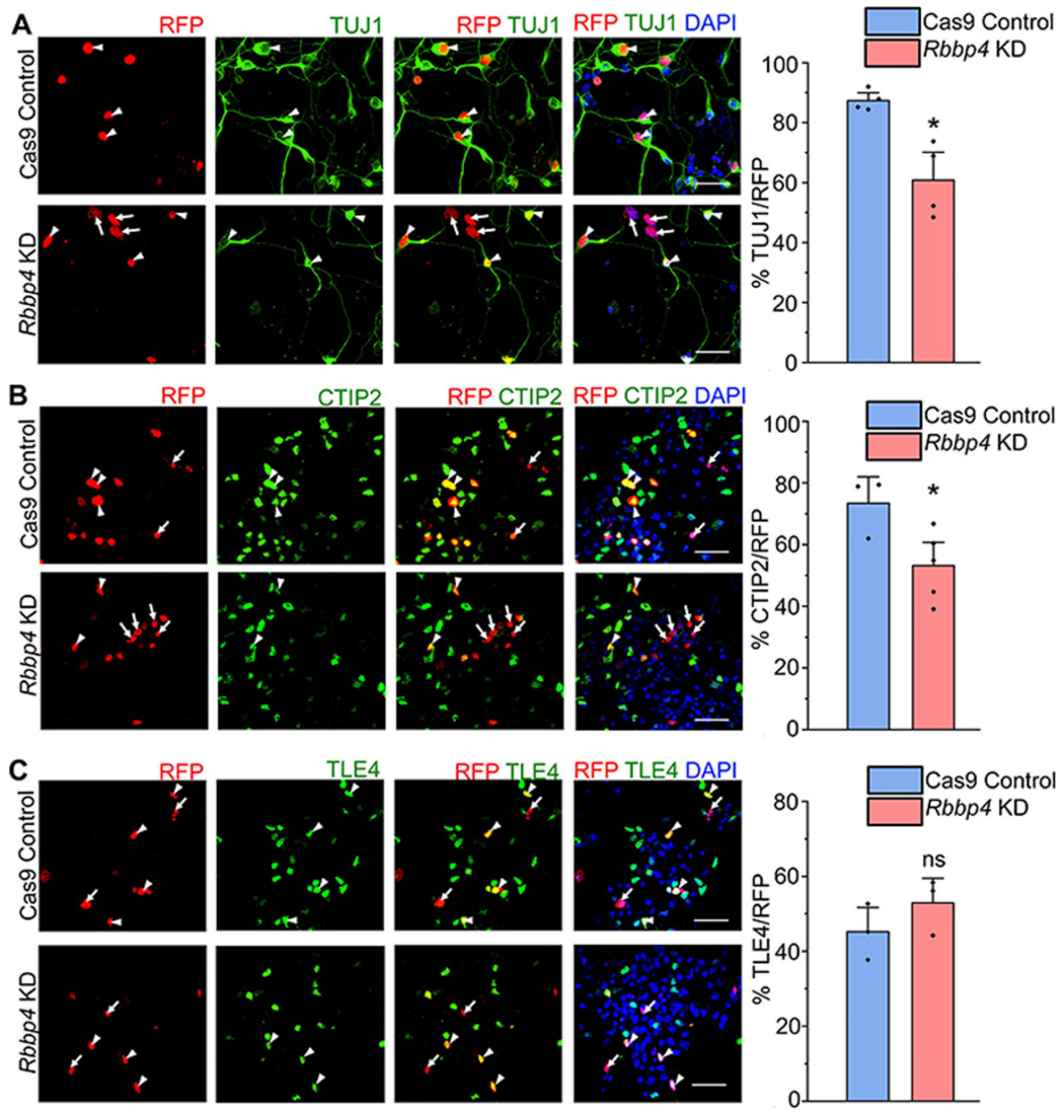
RBBP4 regulates neuronal populations and CTIP2-expressing neurons in the developing neocortical progenitors. ***A***–***C***, Confocal images showing the cultured neocortical cells stained with anti-TUJ1 (***A***), anti-CTIP2 (***B***), and anti-TLE4 (***C***) antibodies after 5DIV and imaged at 60×. Neocortical cells of E12.5 mouse embryo transfected with the Cas9-mCherry-*Rbbp4* sgRNAs vector expressing RFP (left panel of ***A***–***C***) and immunostained with TUJ1 (green, middle panel, ***A***), CTIP2 (green, middle panel, ***B***), and TLE4 (green, middle panel, ***C***) antibody. Right panel of ***A, B***, and ***C*** shows the bar graph with the percentage of TUJ1-positive (***A***), CTIP2-positive (***B***), and TLE4-positive (***C***) cells of the transfected cell populations for the Cas9 control and *Rbbp4* KD neocortical cells. Data are presented as mean ± SEM; two-tailed Student’s *t* test; *p < 0.05 and ns, not significant. White arrowheads indicate transfected cells which express RFP and the indicated markers of panels ***A, B***, and ***C***, and white arrows show transfected cells which express RFP and do not express the indicated markers. Scale bars: ***A***–***C***, 30 μm. Refer to Extended Data Figure 2-1 for more details.

**Figure 3:**
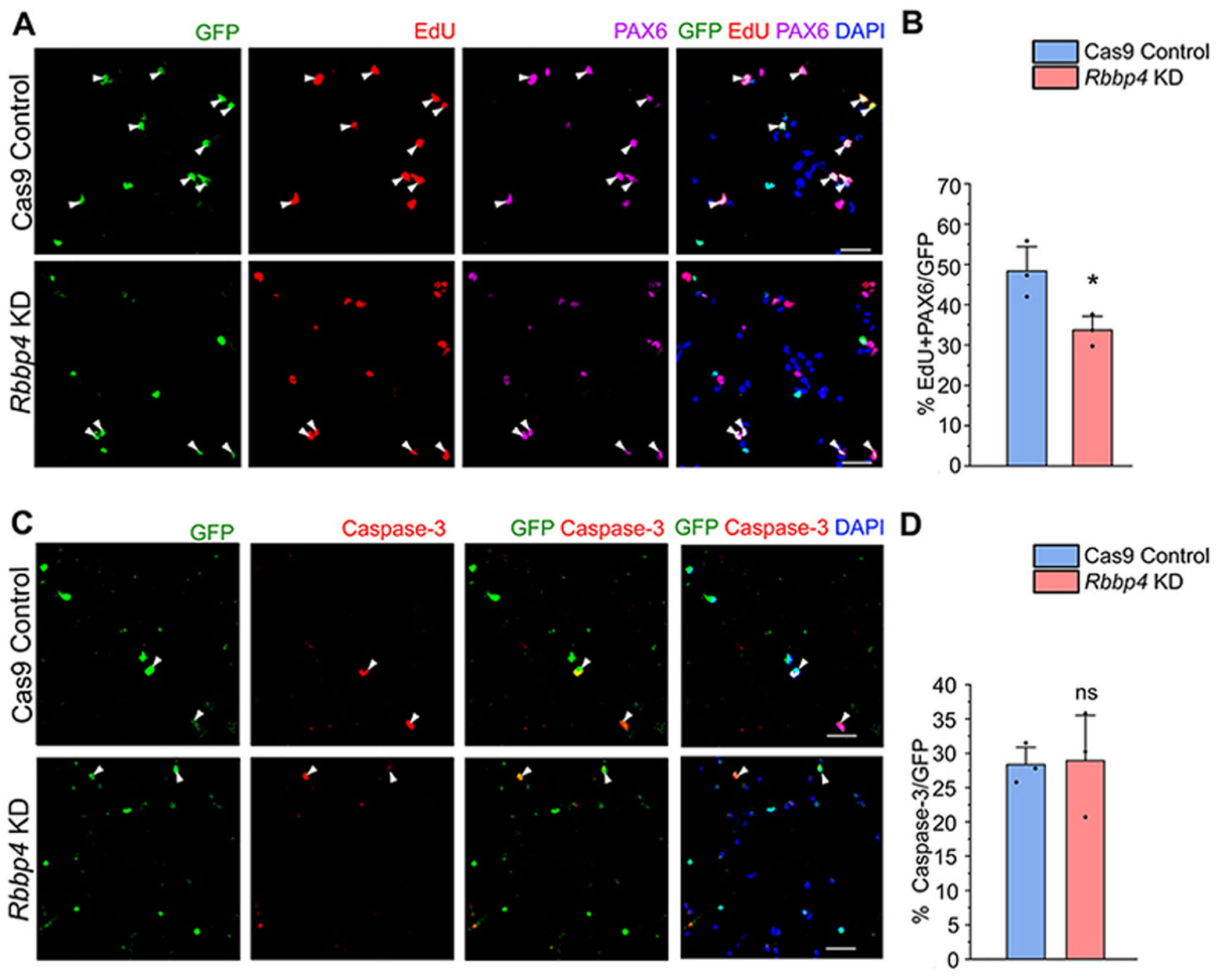
RBBP4 regulates cell proliferation in the cultured neocortical progenitors. Immunostaining images of the neocortical cells of E12.5 mouse embryo transfected with the Cas9 control and *Rbbp4* sgRNAs vector. ***A***, Confocal images showing the transfected neocortical cells incubated with 10 μM EdU for a period of 8 h post 3 h transfection and stained with anti-GFP antibody (green, left panel), EdU (red), and PAX6 (magenta). ***B***, Bar graph showing the percentage of EdU-positive and PAX6-positive cells of the transfected cell populations for the Cas9 control and *Rbbp4* KD neocortical cells. ***C***, Confocal images showing the transfected neocortical cells stained with anti-GFP antibody (green, left panel) and anti-caspase-3 antibody (red, middle panel) after 11 h post transfection and imaged at 60×. ***D***, Bar graph showing the percentage of caspase-3-positive cells of the transfected cell populations for the control and *Rbbp4* KD neocortical cells. Data are presented as mean ± SEM; two-tailed Student’s *t* test; *p < 0.05 and ns, not significant. White arrowheads indicate transfected cells which express GFP and the indicated markers of panels ***A*** and ***C***. Scale bars: ***A, C***, 30 μm.

**Figure 4:**
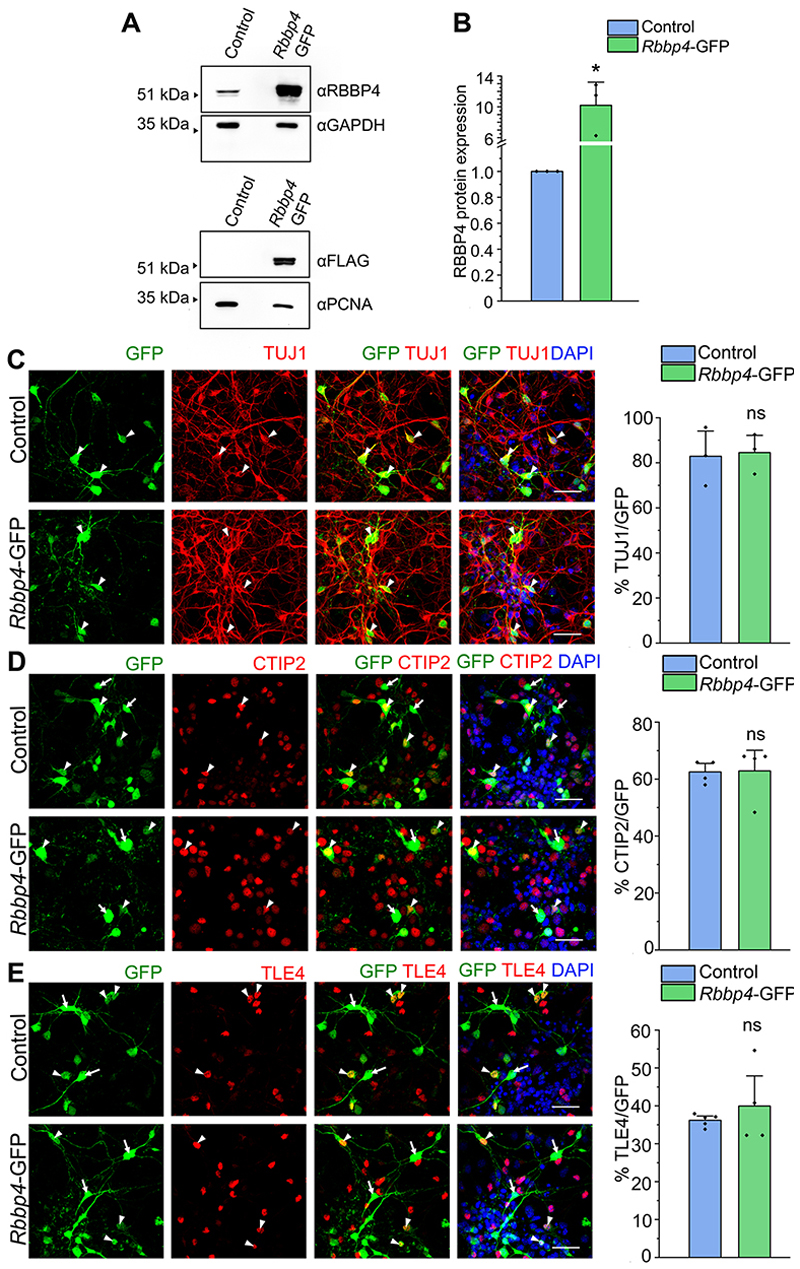
RBBP4 is not sufficient to regulate neuronal fate specification in the neocortical progenitors. ***A***, Western blot showing increased expression of RBBP4 protein levels in the Neuro2A cells transfected with *Rbbp4*-GFP vector (*pCAG-IRES-EGFP*) as compared with the control vector (*pCAG-IRES-EGFP*). Top panel, Immunoblots probed with antibodies against RBBP4 (55 kDa) and GAPDH (37 kDa). Bottom panel, Immunoblots probed with antibodies against FLAG (55 kDa) which is tagged to the RBBP4-ORF in the overexpression constructs and PCNA (28 kDa). Refer to Extended Data Figure 4-1 for more details. ***B***, Bar graph showing the RBBP4 protein levels in the control and *Rbbp4*-GFP transfected Neuro2A cells. Fold changes were normalized to GAPDH levels for each sample. Data are presented as mean ± SEM; two-tailed Student’s *t* test; *p < 0.05. ***C***–***E***, Immunocytochemistry of neocortical progenitor culture transfected with *pCAG-IRES-EGFP* vector (Control) or *pCAG-IRES-EGFP* vector containing the *Rbbp4-*ORF (*Rbbp4-GFP*) at E12.5 and cultured for 5DIV. Confocal images showing the cultured neocortical cells stained with anti-TUJ1 (***C***), anti-CTIP2 (***D***), and anti-TLE4 (***E***) antibodies and imaged at 60×. Neocortical cells transfected with the *Rbbp4-GFP* vector expressing GFP (left panel of ***C***–***E***) and immunostained with TUJ1 (red, middle panel, ***C***), CTIP2 (red, middle panel, ***D***), and TLE4 (red, middle panel, ***E***) antibody. The right panel of ***C***–***E*** shows the bar graph with the percentage of TUJ1-positive (***C***), CTIP2-positive (***D***), and TLE4-positive (***E***) cells of the transfected cell populations for the control and *Rbbp4-GFP* neocortical cells. Data are presented as mean ± SEM; two-tailed Student’s *t* test for panel ***C*** and ***E***; Mann–Whitney test (two tailed) for panel ***D*** and ns, not significant. White arrowheads indicate transfected cells which express GFP and the indicated markers of panels ***C***–***E***, and white arrows show transfected cells which express GFP and do not express the indicated markers. Scale bars: ***C***–***E***, 30 μm.

**Figure 5:**
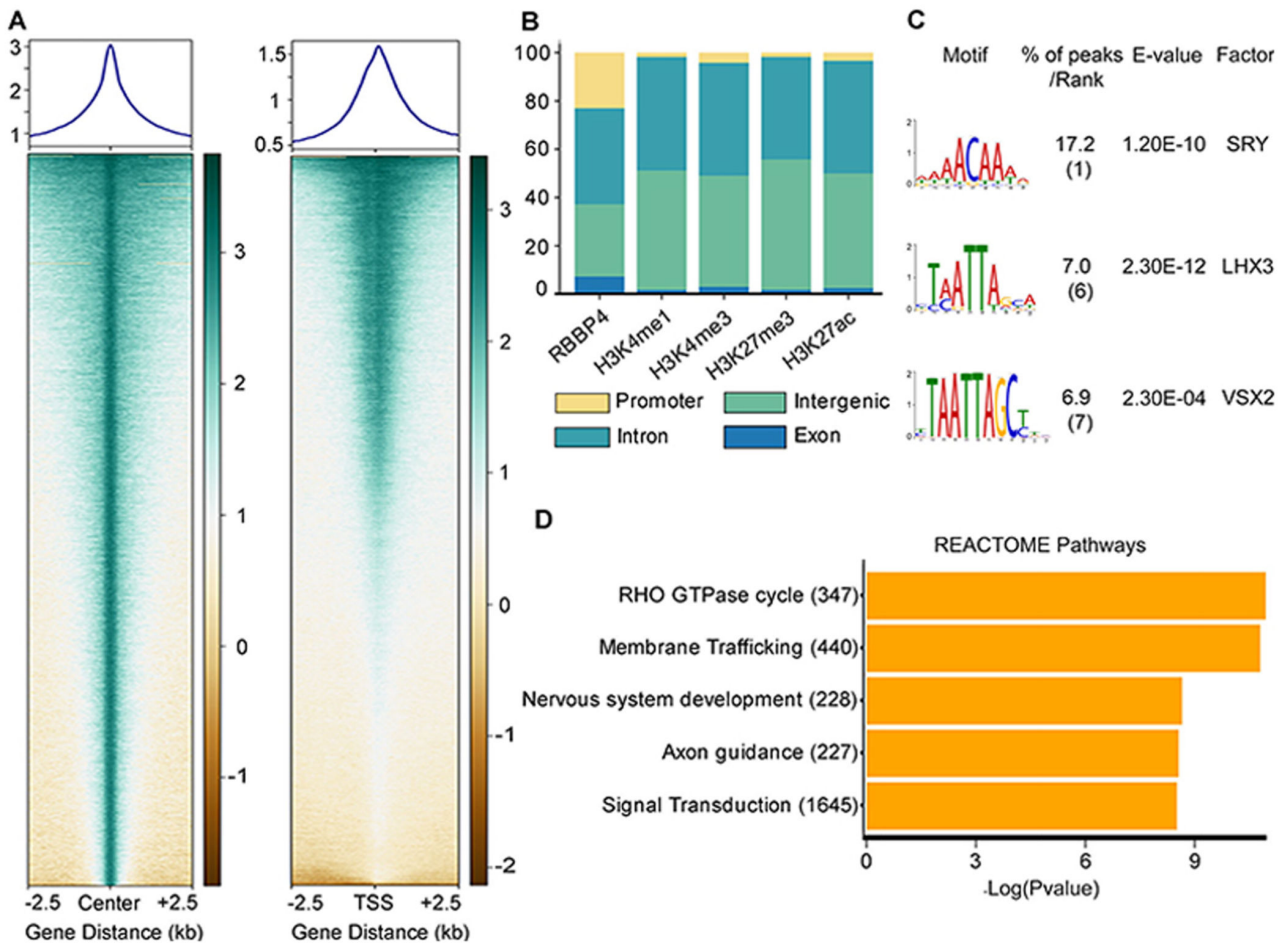
Identification of RBBP4 genome-wide occupancy in the developing neocortex by ChIP sequencing. ***A***, Heatmap plot showing the genome occupancy of RBBP4 over regions within 2.5 upstream and downstream of peak centers (left panel) and TSS (right panel). The gradient green-to-yellow color indicates high-to-low counts in the corresponding regions. ***B***, Stacked bar plot showing the genomic distribution of called peaks for RBBP4 ChIP-seq experiments along with histone marks previously reported by Sun et al. (2015). Refer to Extended Data Figure 5-1 for more details. ***C***, MEME plot showing the most significantly enriched DNA motifs identified in RBBP4 ChIP-seq peaks. Refer to Extended Data Figure 5-2 and Table 5-1 for more details. ***D***, REACTOME pathways plot showing the top 5 most significant REACTOME pathways terms of all RBBP4-bound genes. The *x*-axis indicates the significance of indicated pathway ranked by −log 10 *p* value and the *y*-axis indicates the names of REACTOME pathways. Refer to Extended Data Table 5-2 for more details.

**Figure 6:**
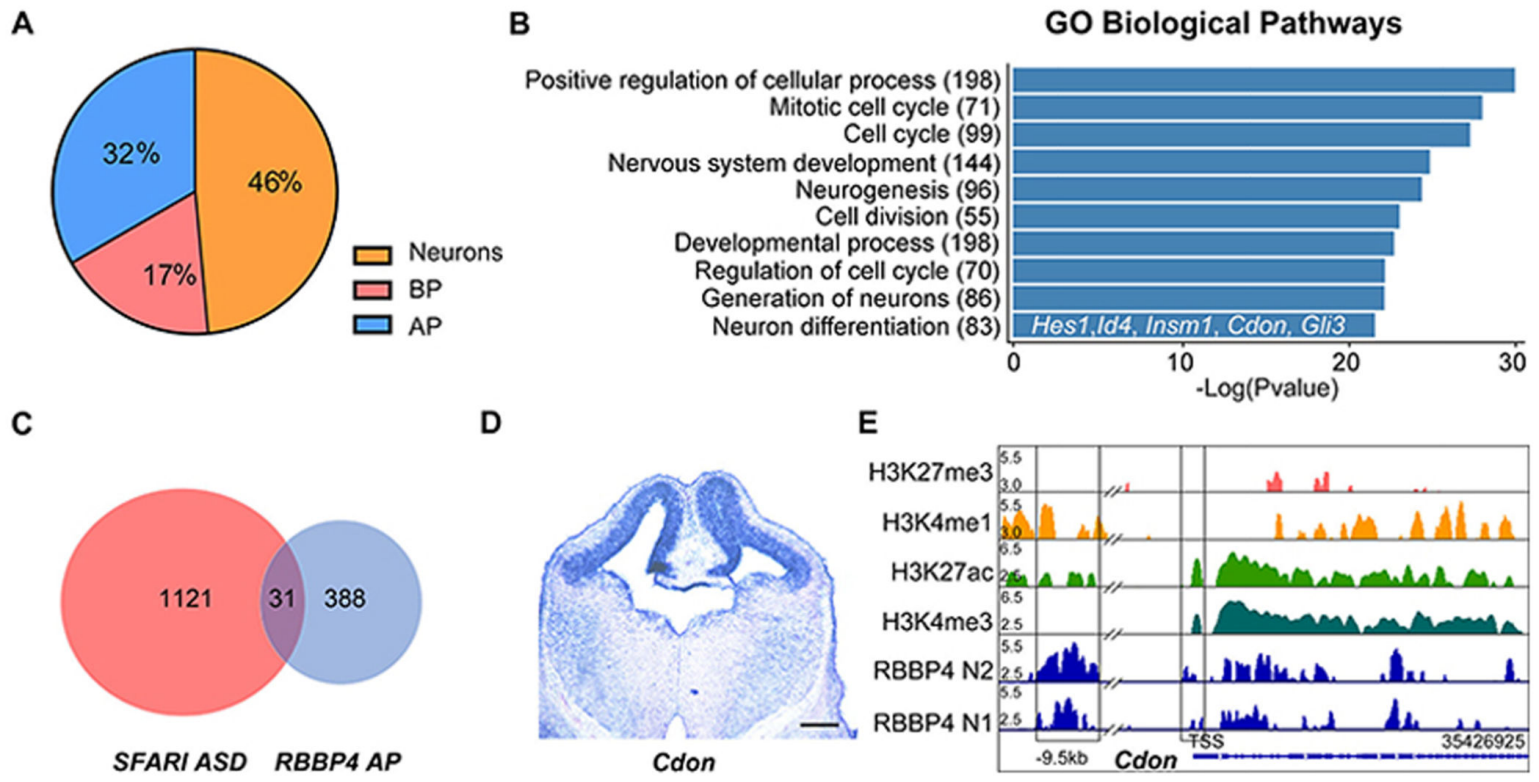
CDON is identified to be a novel RBBP4-bound gene. ***A***, Pie chart showing the RBBP4-occupied genes enriched in different cortical cell types: AP, apical progenitors; BP, basal progenitors and neurons (data from Telley et al., 2019). Refer to Extended Data Table 6-1 for more details. ***B***, Gene Ontology (GO) analysis of the biological process showing the top 10 enriched pathways common between RBBP4 and apical progenitor genes (Telley et al., 2019) ranked by −log 10 *p* value. Refer to Extended Data Table 6-2 for more details. ***C***, Pie chart showing the autism-associated genes within the apical progenitor-specific RBBP4-bound loci (our ChIP-seq data compared with data from Safari gene database; https://gene.sfari.org). Refer to Extended Data Table 6-3 for more details. ***D***, ISH images showing the expression of *Cdon* in the neocortex of a mouse embryo at E12.5. Scale bar, 100 μm (refer to Extended Data Fig. 6-1 for more details). ***E***, IGV genome browser plots of the *Cdon* locus showing binding of RBBP4, along with histone marks H3K4me3, H3K27ac, H3K4me1, and H3K27me3 (data from our ChIP-seq analysis and Sun et al., 2015). Refer to Extended Data Figure 6-4 for more details.

**Figure 7:**
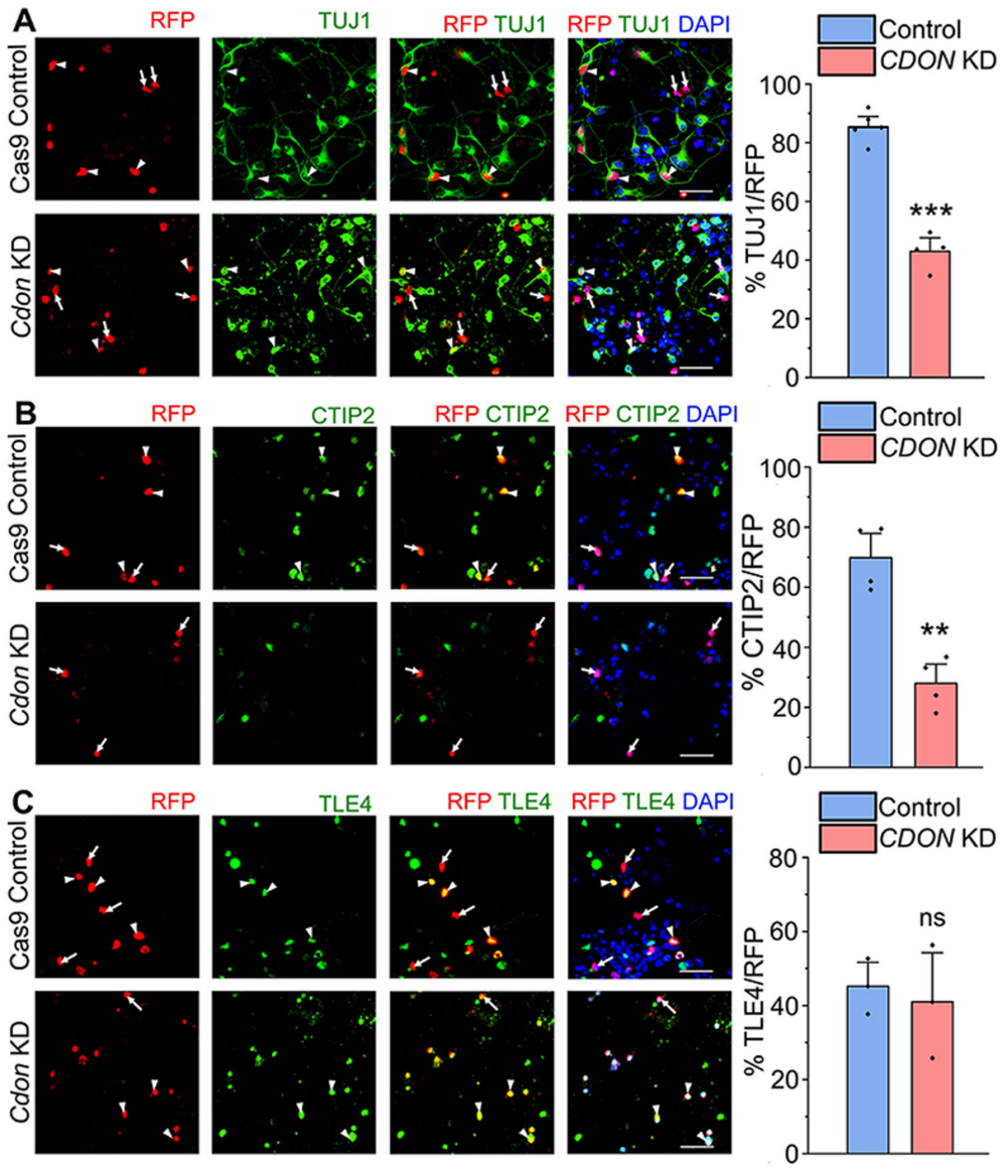
CDON regulates neurogenesis and CTIP2-expressing neurons in the neocortical progenitors. ***A***, Neocortical progenitors of E12.5 mouse embryo transfected with the Cas9-*Cdon* sgRNAs vector (left panel of ***A***–***C***) and immunostained with TUJ1 (green, middle panel, ***A***), CTIP2 (green, middle panel, ***B***), and TLE4 (green, middle panel, ***C***) antibody. The right panel of ***A***–***C*** shows the bar graph with the percentage of TUJ1-positive (***A***), CTIP2-positive (***B***), and TLE4-positive (***C***) cells of the transfected cell populations for the Cas9 control and *Cdon* KD neocortical cells. Data are presented as mean ± SEM; two-tailed Student’s *t* test; **p < 0.0, ***p < 0.001, and ns, not significant. White arrowheads indicate transfected cells which express RFP and the indicated markers of panels ***A***–***C***, and white arrows show transfected cells which express RFP and do not express the indicated markers. Scale bars: ***A***–***C***, 30 μm. Refer to Extended Data Figures 7-1 and 7-2 for more details.

**Figure 8:**
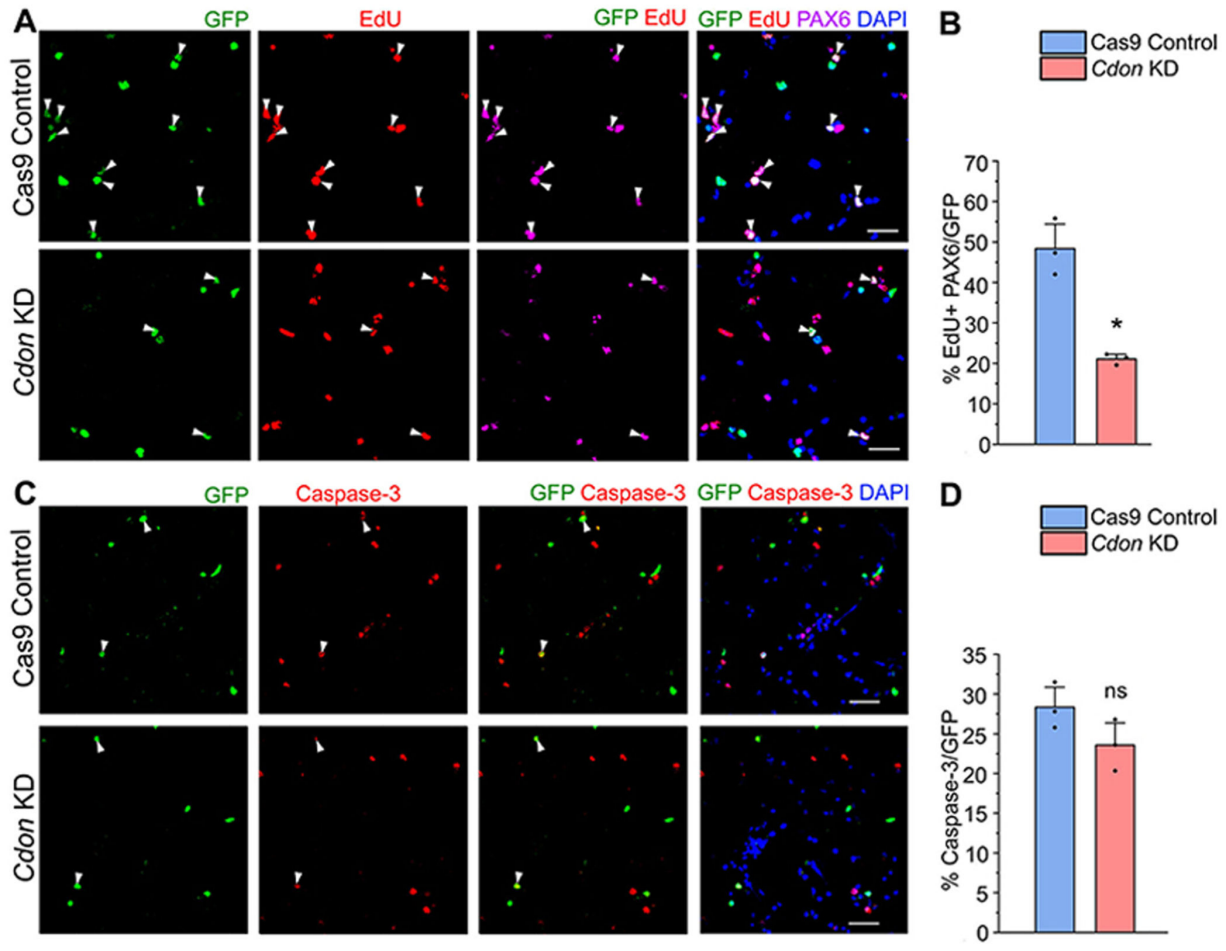
CDON regulates cell proliferation in the neocortical progenitors. Neocortical progenitor culture of E12.5 mouse embryo is transfected with the Cas9 control and *Cdon* sgRNAs vector. ***A***, Confocal images show the transfected neocortical cells incubated with a pulse of 10 μM EdU for 8 h after 3 h of transfection and immunostained with anti-GFP antibody (green, left panel) and EdU (red, middle panel) and imaged at 60×. ***B***, Bar graph showing the percentage of EdU-positive and PAX6-positive cells of the transfected cell populations for the Cas9 control and *CDON* KD neocortical cells. ***C***, Confocal images showing the transfected neocortical cells stained with anti-GFP antibody (green, left panel) and anti-caspase-3 antibody (red, middle panel) after 11 h post transfection and imaged at 60×. ***D***, Bar graph with the percentage of caspase-3-positive cells of the transfected cell populations for the control and *Cdon* KD neocortical cells. Data are presented as mean ± SEM; two-tailed Student’s *t* test; *p < 0.05 and ns, not significant. White arrowheads indicate transfected cells which express GFP and the indicated markers of panels ***A*** and ***C***. Scale bars: ***A*** and ***C***, 30 μm.

**Figure 9:**
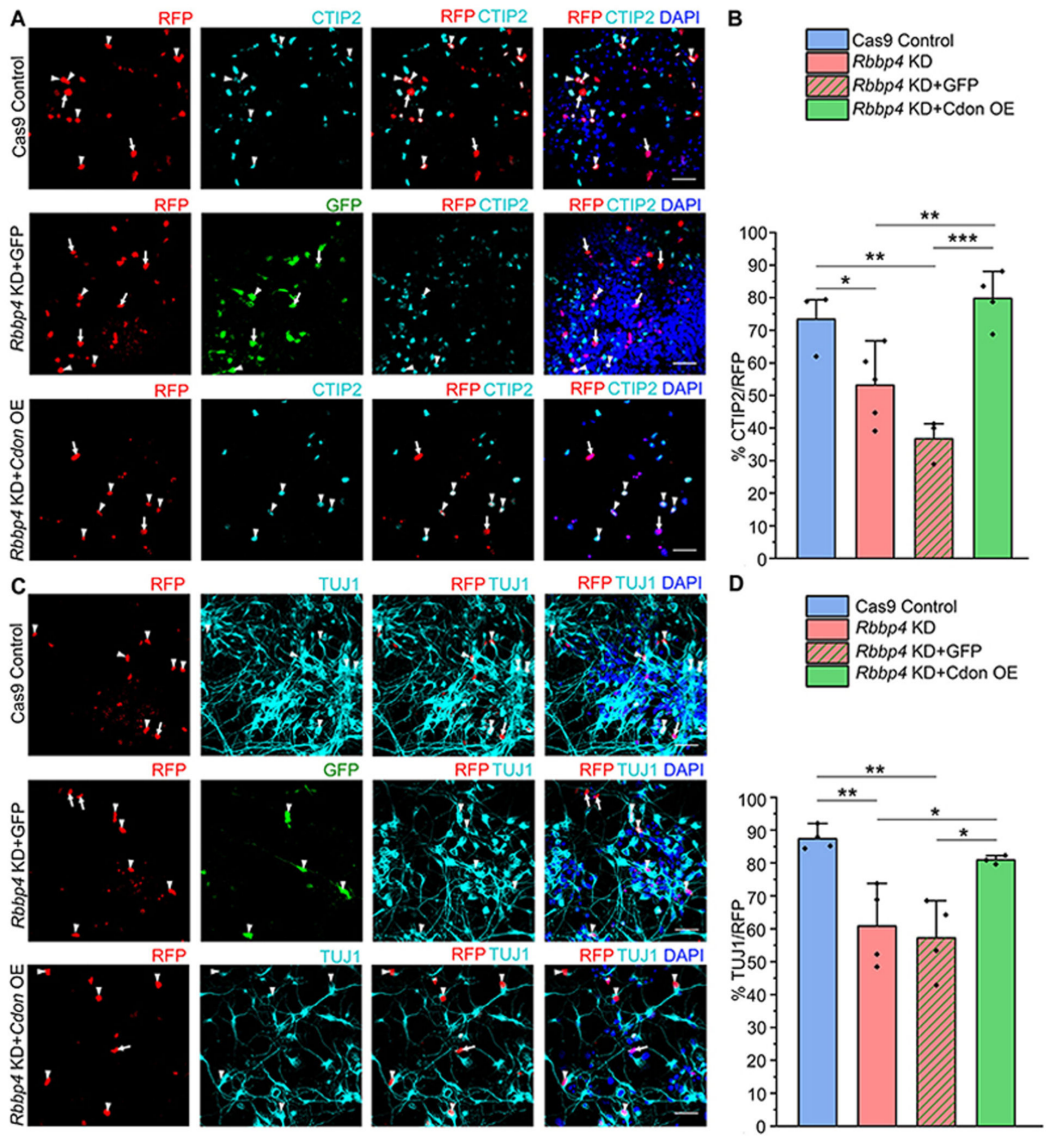
Overexpression of CDON rescues the phenotype caused by loss of RBBP4 in neocortical progenitors. Immunostained neocortical progenitors of E12.5 mouse embryo transfected with the Cas9 control, *Rbbp4* gRNAs along with either pCAG-IRES-EGFP (RBBP4 KD-GFP) or pEF1α-*Cdon* expressing vector (RBBP4 KD-CDON OE). ***A, C***, Confocal images showing the cultured neocortical progenitors stained with anti-CTIP2 (***A***) and anti-TUJ1 (***B***) antibodies after 5DIV. ***B, D***, Bar graph showing the percentage of CTIP2-positive (***B***) and TUJ1-positive (***D***) cells of the RFP transfected cell populations for the Cas9 control, *Rbbp4* KD, *Rbbp4* KD-GFP, and RBBP4 KD-CDON OE neocortical progenitors. Data are presented as mean ± SEM; one-way ANOVA, followed by Tukey’s multiple-comparisons test, *p < 0.05, **p < 0.01, and ***p < 0.001. White arrowheads indicate transfected cells which express RFP and the indicated markers of panels ***A*** and ***C***, and white arrows show transfected cells which express RFP and do not express the indicated markers. Scale bars: ***A*** and ***C***, 30 μm.

**Figure 10:**
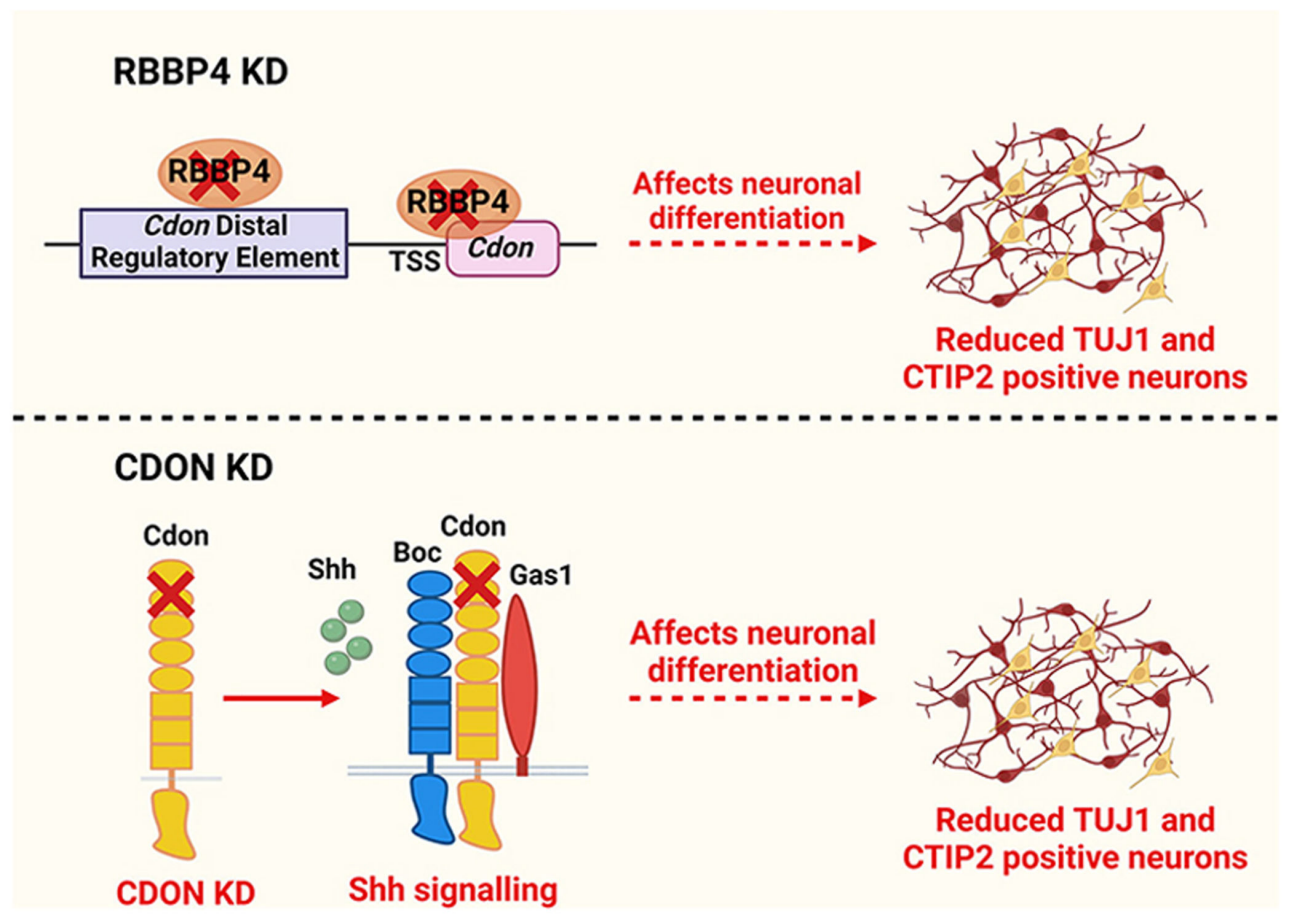
RBBP4 regulates neurogenesis in the neocortical progenitors. RBBP4 binds to the gene regulatory elements of *Cdon* and *Rbpp4* knockdown reduces the neuronal numbers specifically, CTIP2-expressing neurons. *Cdon* knockdown phenocopies *Rbbp4* knockdown and can rescue Rbbp4 loss and is a novel regulator of neurogenesis in the neocortical progenitors. Image made using BioRender.com.

**Table 1:** Primers used for generating probes for RNA-in situ hybridization

Gene name	Primer sequence (5′>3′)	
*Rbbp4* antisense	Forward	AGAACGGGTGATCAACGAGG
*Rbbp4* antisense	Reverse	TGTGAGCATCAACCGAGTGG
*Rbbp4* sense	Forward	AGAACGGGTGATCAACGAGG
*Rbbp4* sense	Reverse	TGTGAGCATCAACCGAGTGG
Cdon antisense	Forward	TAAGCGGAGGCCTGG
Cdon antisense	Reverse	ATTCGAGGAAGGACGACTC
Cdon sense	Forward	TAAGCGGAGGCCTGG
Cdon sense	Reverse	ATTCGAGGAAGGACGACTC

Sequences of primers for generating probes of *Rbbp4* and *Cdon* for RNA-in situ hybridization experiments are listed in the table. Primers were designed using Primer 3 (http://bioinfo.ut.ee/primer3-0.4.0/).

**Table 2:** Primers used for amplifying *Rbbp4* gene for the construct *RBBP4*-GFP

Primer name	Primer sequence
*Rbbp4* forward primer	5′ GTCTCATCATTTTGGCAAAGAATTCATG**GACTACAAAGACGATGACGACAAG**GCTGACAAGGAAGCGGCCTTTGACGACGCAGTGG 3’
*Rbbp4* reverse primer	5′ GTACCGTCGACTGCAGATTTAAATCTAGGACCCTTGTCCCTC 3’

Primer sequences for amplifying *RBBP4* ORF for the construct RBBP4-GFP. The forward primer contained a FLAG-tag sequence (marked in bold letters) inserted between the ATG and the start of the gene.

**Table 3:** Primers used for quantitative real-time PCR

Gene name	Primer sequence (5′>3′)	
*Cdon*	Forward	GAGACGTTGTGGAAGGCTCA
	Reverse	TGCTGAACTCACTCTCCCCT
*Gapdh*	Forward	CTTTGGCATTGTGGAAGGGC
	Reverse	TGCAGGGATGATGTTCTGGG

Sequences of primers of *Cdon* and *Gapdh* used for quantitative real-time PCR are listed in the table. Primers were designed using Primer 3 (http://bioinfo.ut.ee/primer3-0.4.0/).

## Data Availability

The ChIP-seq data associated with this manuscript has been submitted to Sequence Read Archive (SRA) with project ID PRJNA942063.

## Significance Statement

Chromatin modifier RBBP4 regulates chromatin structure and, thereby, gene expression. It is expressed in the dorsal telencephalon progenitors during deeplayer neurogenesis. In this study, we unveil a novel role for RBBP4 in regulating deeplayer neurogenesis in the neocortical progenitors. Our research underscores RBBP4’s critical role in governing progenitor proliferation and neuronal subtype specification in the neocortex while identifying its genome-wide binding occupancy profile. Moreover, we identify Cdon as a novel binding target of RBBP4, also involved in regulating deep-layer neurogenesis. These findings illuminate the mechanisms by which chromatin modifiers influence neocortical development, offering insights into how mutations in chromatin modifiers could impact cortical development and contribute to neurodevelopmental disorders.
