# Supplementary figures and images for: Histone-Binding Protein RBBP4 Is Necessary to Promote Neurogenesis in the Developing Mouse Neocortical Progenitors
Source: eNeuro. Author manuscript; Available in PMC 2025 May 19. (PMC7617683; doi:10.1523/ENEURO.0391-23.2024)

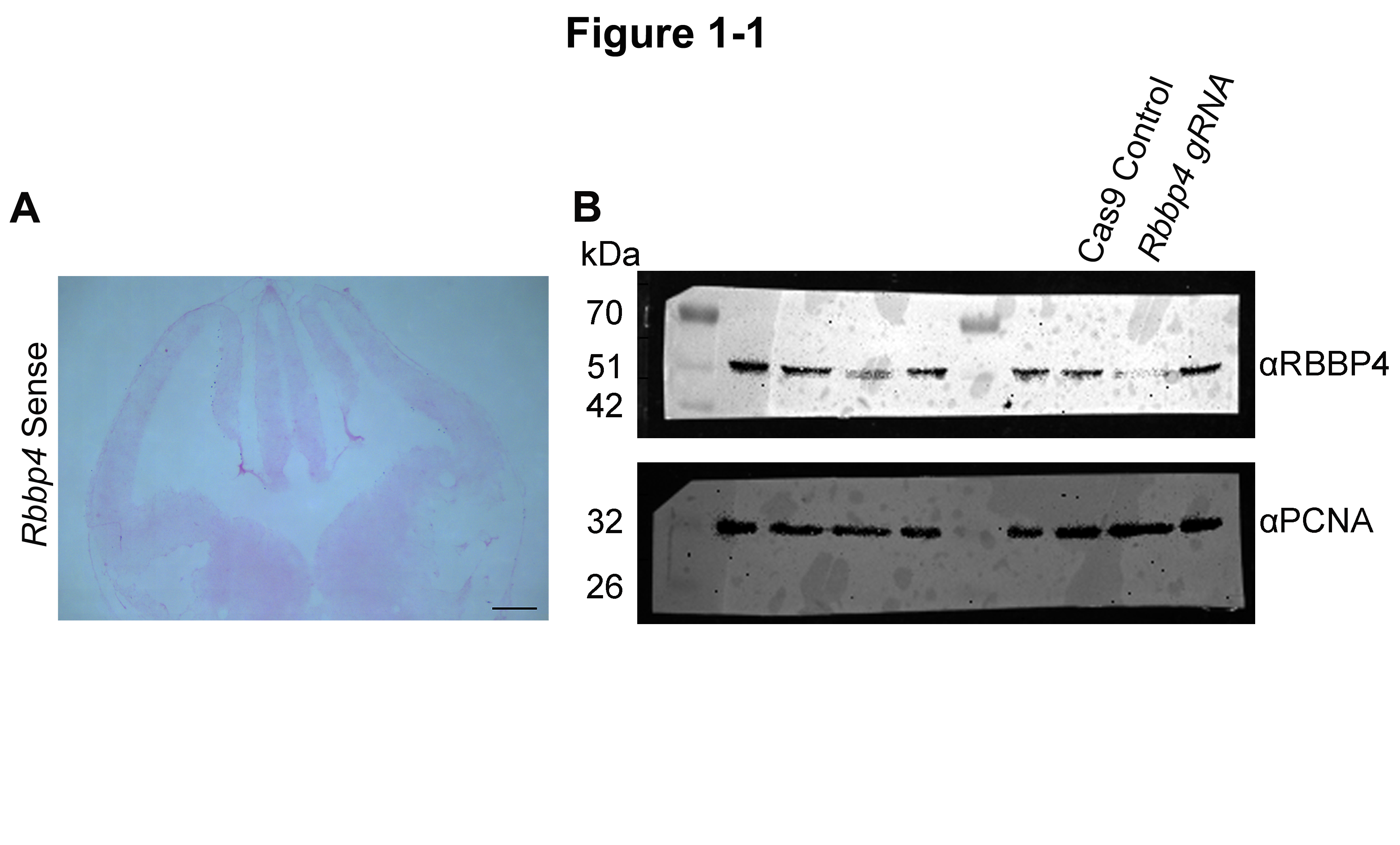

Supplement: Supplementary Figure 1-1 [file EMS205222-supplement-Supplementary_Figure_1_1.tif]

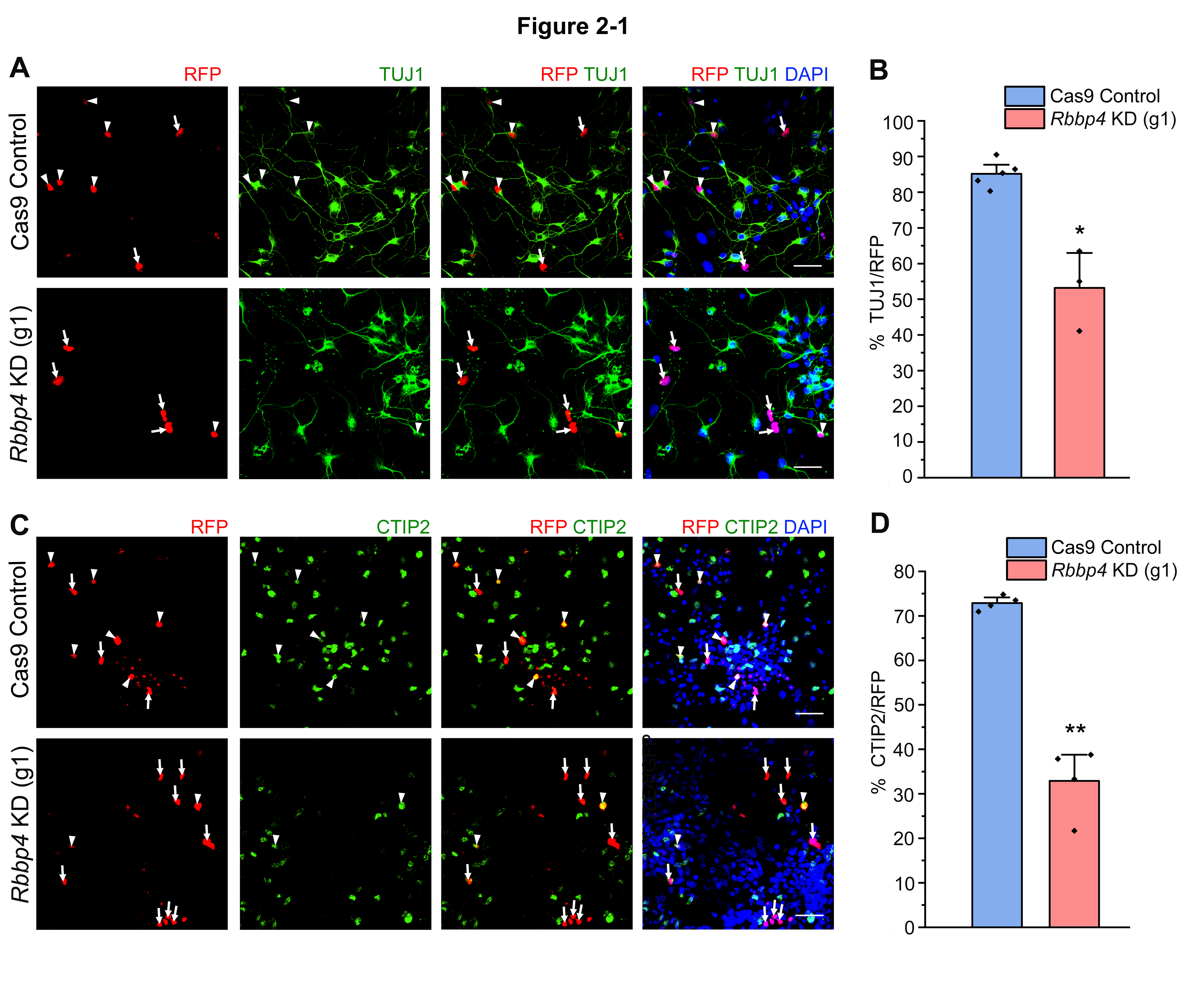

Supplement: Supplementary Figure 2-1 [file EMS205222-supplement-Supplementary_Figure_2_1.tif]

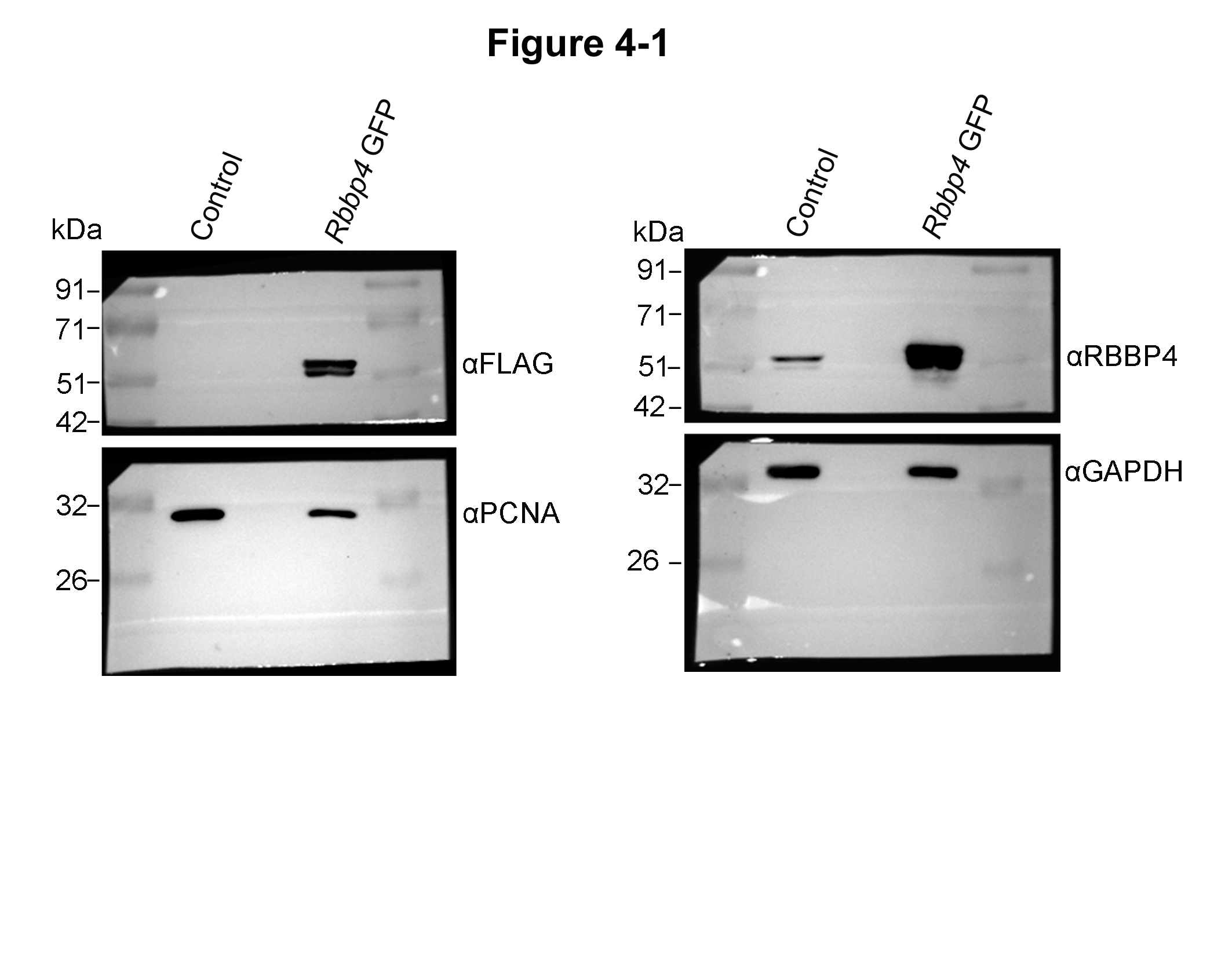

Supplement: Supplementary Figure 4-1 [file EMS205222-supplement-Supplementary_Figure_4_1.tif]

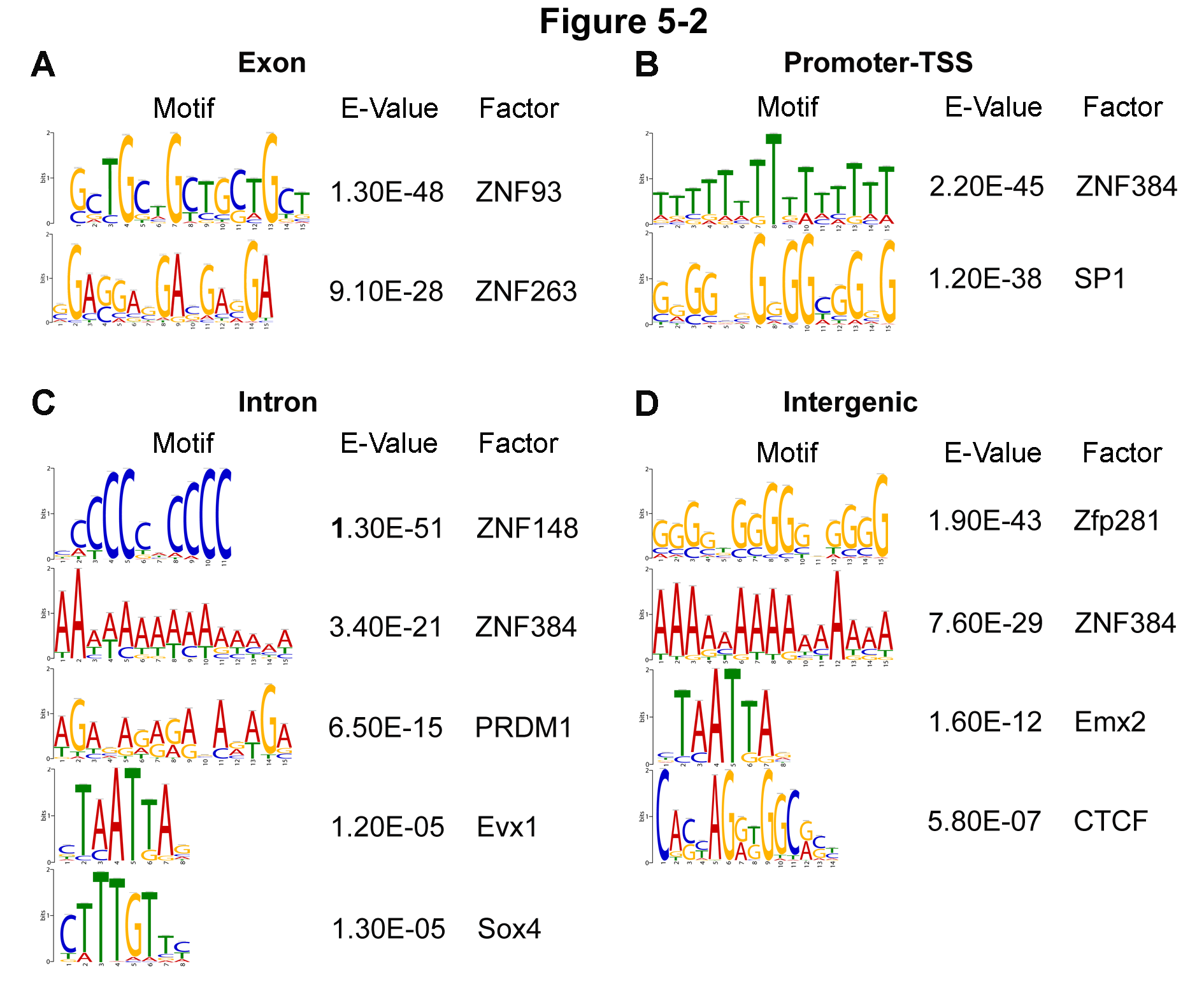

Supplement: Supplementary Figure 5-2 [file EMS205222-supplement-Supplementary_Figure_5_2.tif]

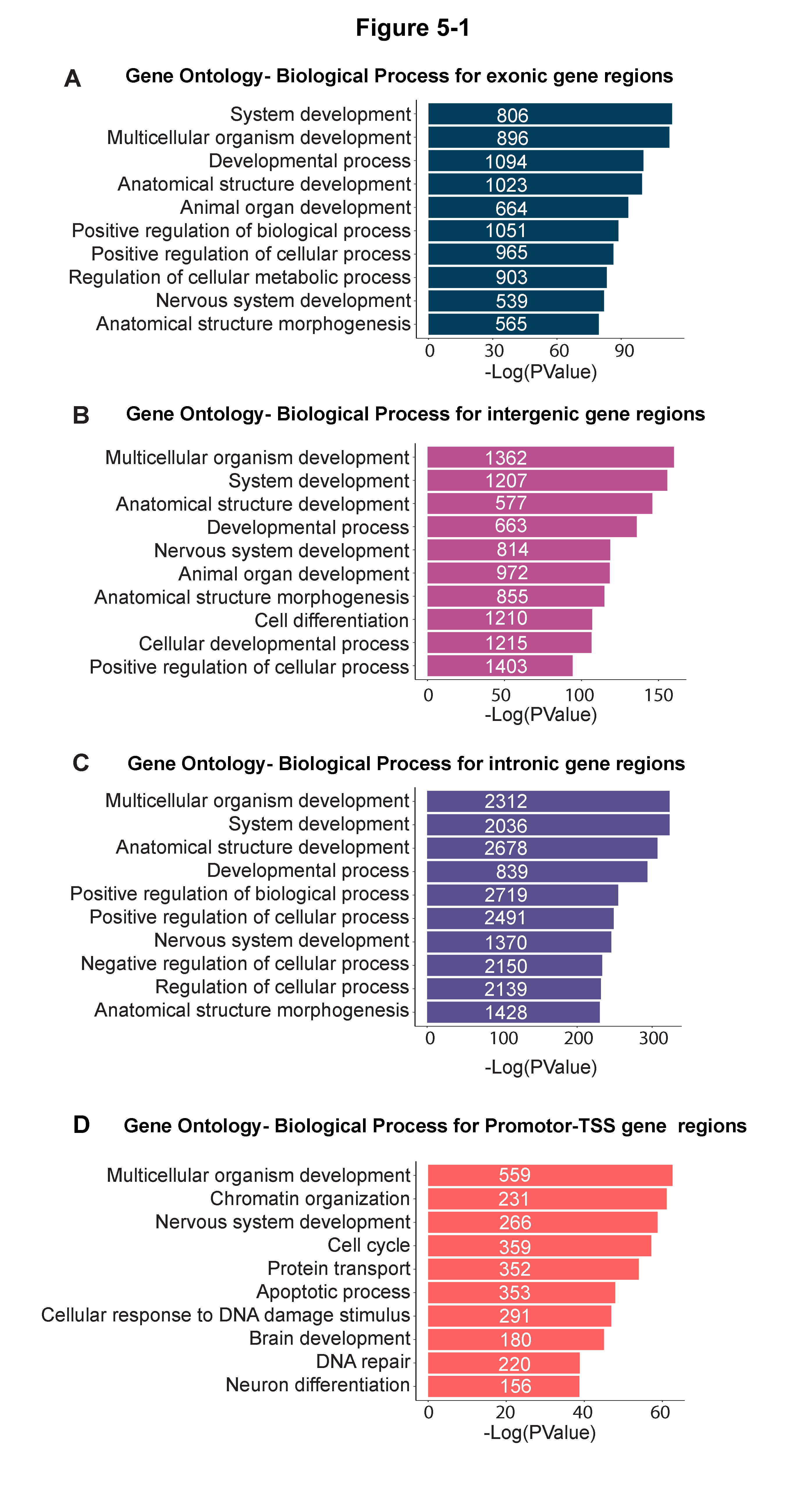

Supplement: Supplementary Figure 5-1 [file EMS205222-supplement-Supplementary_Figure_5_1.tif]

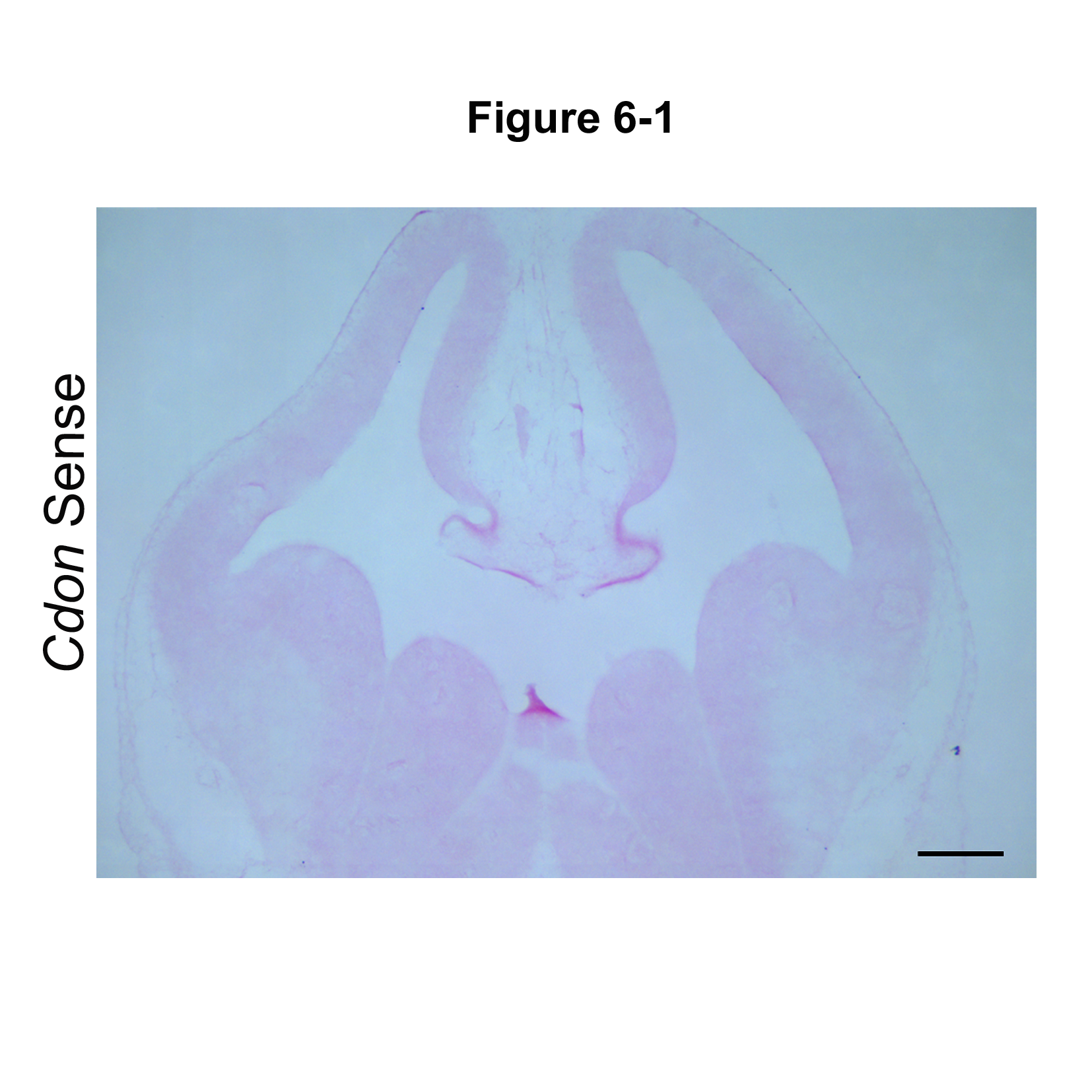

Supplement: Supplementary Figure 6-1 [file EMS205222-supplement-Supplementary_Figure_6_1.tif]

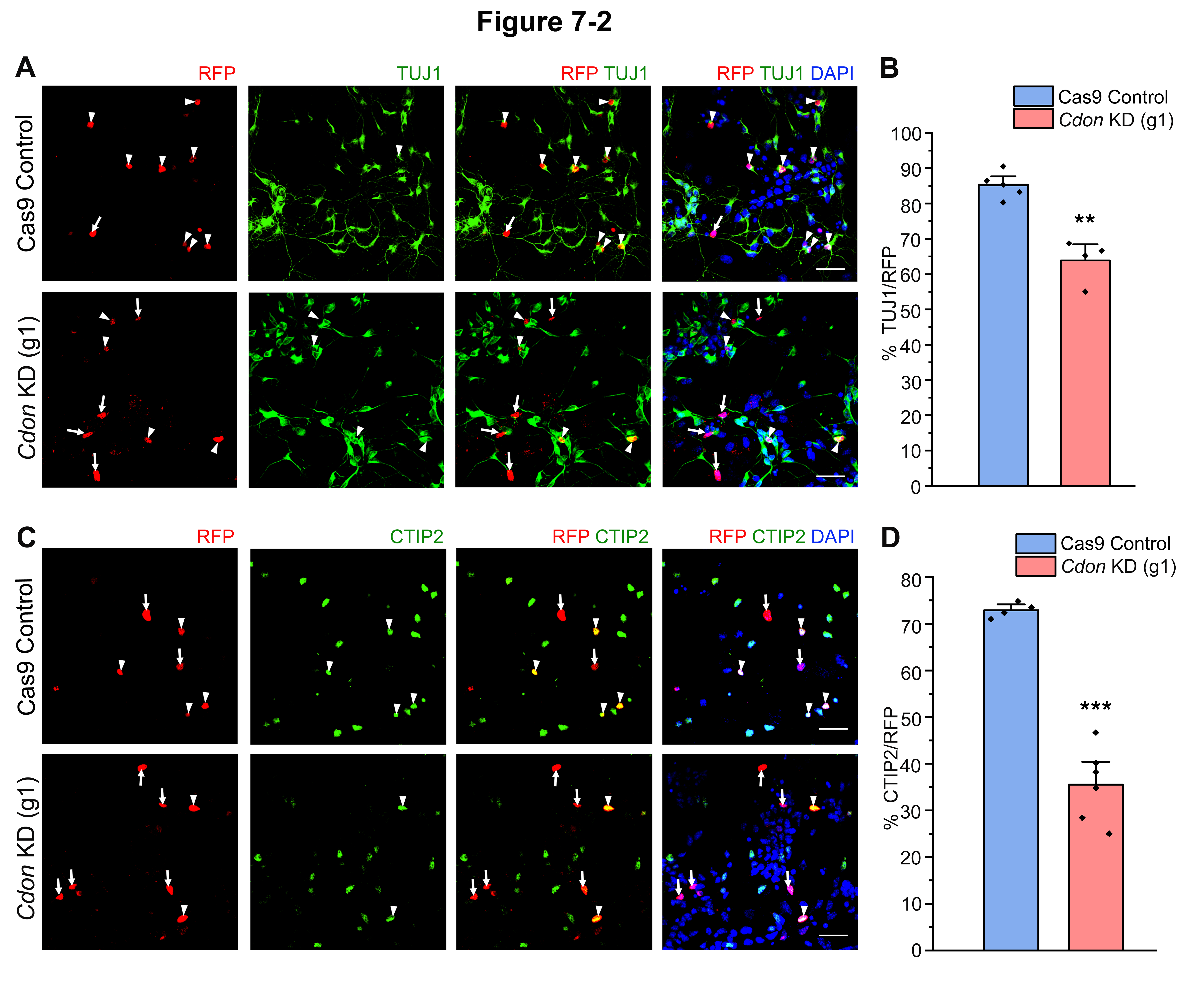

Supplement: Supplementary Figure 7-1 [file EMS205222-supplement-Supplementary_Figure_7_1.tif]

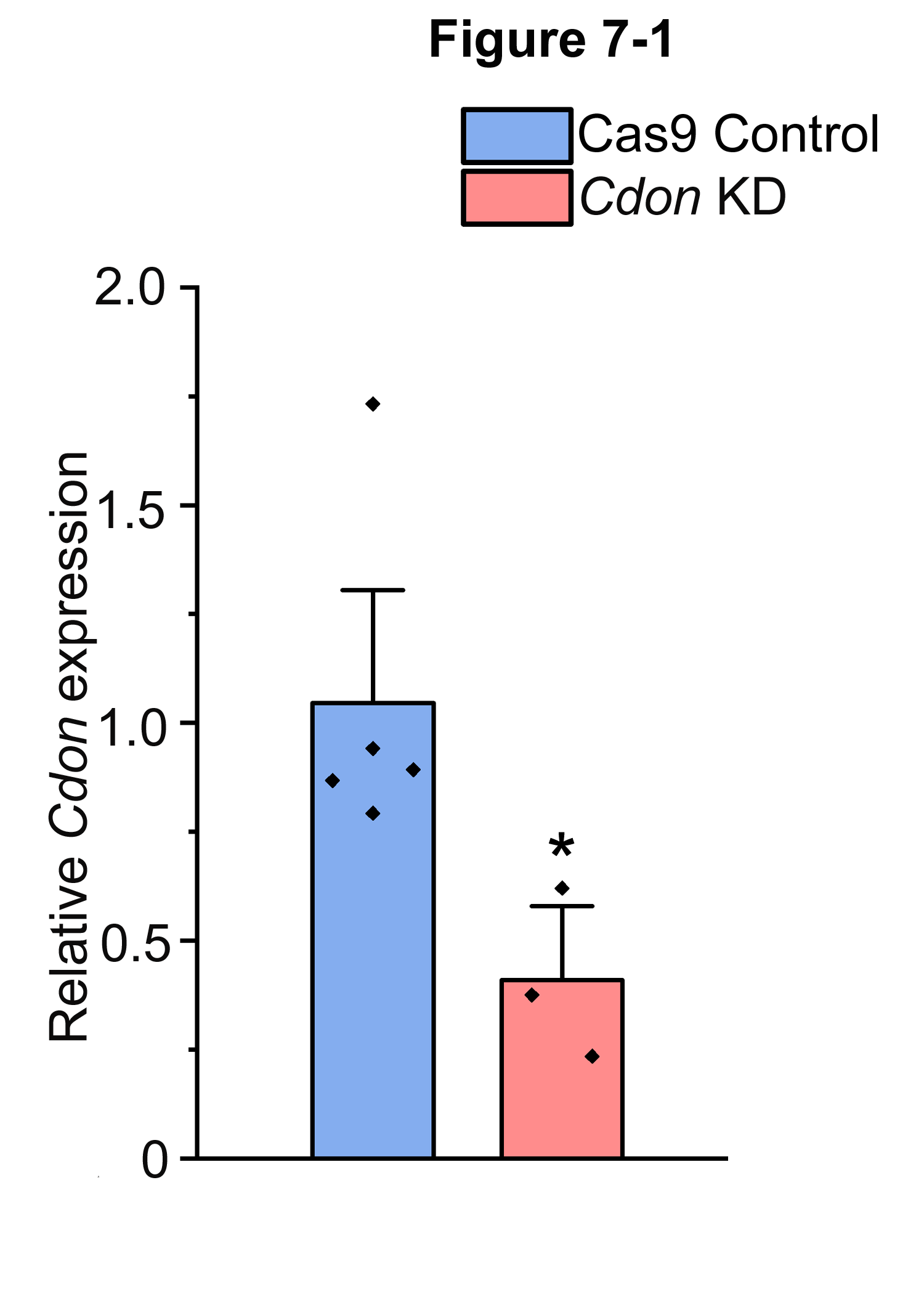

Supplement: Supplementary Figure 7-2 [file EMS205222-supplement-Supplementary_Figure_7_2.tif]
